# Supplementary material for: A bioinformatics approach to identifying Wolbachia infections in arthropods
Source: PeerJ. 2018 Sep 3;6:e5486. doi: 10.7717/peerj.5486 (PMC6126470; doi:10.7717/peerj.5486)
Supplement: Supplemental Information 1 — Supplementary figures/tables and code used for data analysis [file peerj-06-5486-s001.zip › Supplement/Pascar Chandler 2018 Supplementary Figures.docx]

Figure S1. Molecular Phylogenetic analysis by Maximum Likelihood method of the *ftsZ* gene. The evolutionary history was inferred by using the Maximum Likelihood method based on the Tamura 3-parameter model (Tamura, 1992). The tree with the highest log likelihood (-1923.41) is shown. The percentage of trees in which the associated taxa clustered together is shown next to the branches. Initial tree(s) for the heuristic search were obtained automatically by applying Neighbor-Join and BioNJ algorithms to a matrix of pairwise distances estimated using the Maximum Composite Likelihood (MCL) approach, and then selecting the topology with superior log likelihood value. A discrete Gamma distribution was used to model evolutionary rate differences among sites (5 categories (+G, parameter = 0.2034)). The tree is drawn to scale, with branch lengths measured in the number of substitutions per site. The analysis involved 53 nucleotide sequences. There were a total of 701 positions in the final dataset. Evolutionary analyses were conducted in MEGA7 (Kumar et al., 2016). Isolates of known *Wolbachia* Supergroups are marked with a ◼. Species that have not previously been identified to harbor *Wolbachia* infections are marked with a ▲.

*
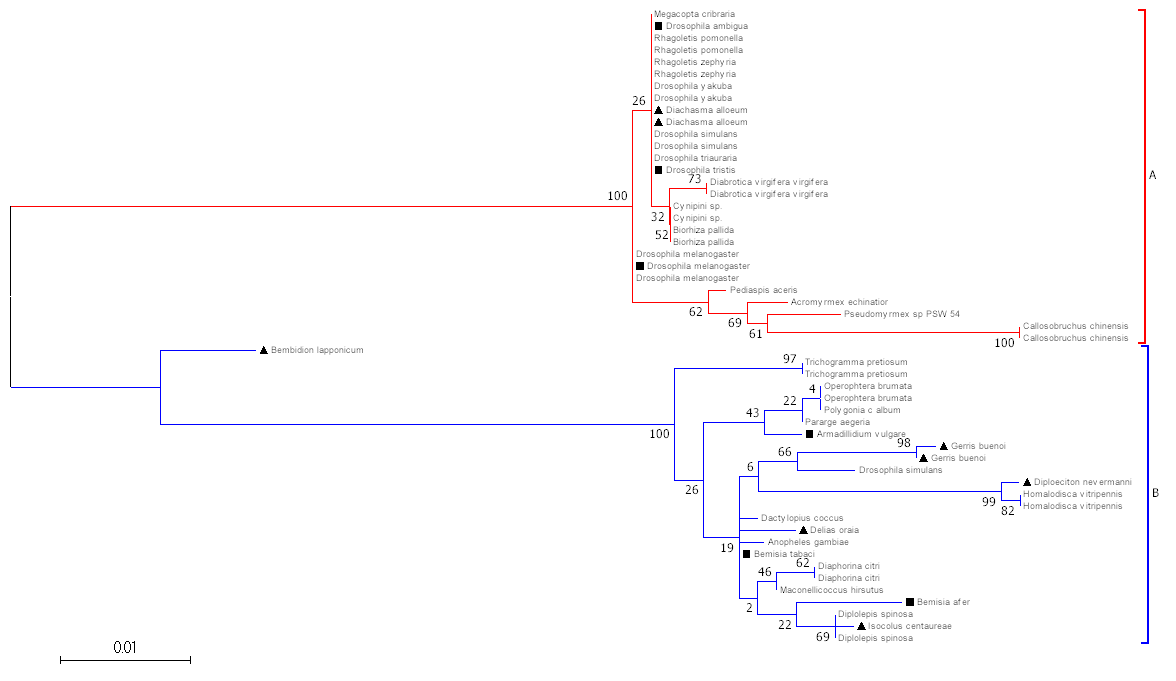
*

Figure S2. Molecular Phylogenetic analysis by Maximum Likelihood method of the *groE* operon. The evolutionary history was inferred by using the Maximum Likelihood method based on the Hasegawa-Kishino-Yano model (Hasegawa et al., 1985). The tree with the highest log likelihood (-2339.28) is shown. The percentage of trees in which the associated taxa clustered together is shown next to the branches. Initial tree(s) for the heuristic search were obtained automatically by applying Neighbor-Join and BioNJ algorithms to a matrix of pairwise distances estimated using the Maximum Composite Likelihood (MCL) approach, and then selecting the topology with superior log likelihood value. A discrete Gamma distribution was used to model evolutionary rate differences among sites (5 categories (+G, parameter = 0.5509)). The tree is drawn to scale, with branch lengths measured in the number of substitutions per site. The analysis involved 53 nucleotide sequences. There were a total of 717 positions in the final dataset. Evolutionary analyses were conducted in MEGA7 (Kumar et al., 2016). Isolates of known *Wolbachia* Supergroups are marked with a ◼. Species that have not previously been identified to harbor *Wolbachia* infections are marked with a ▲.

*
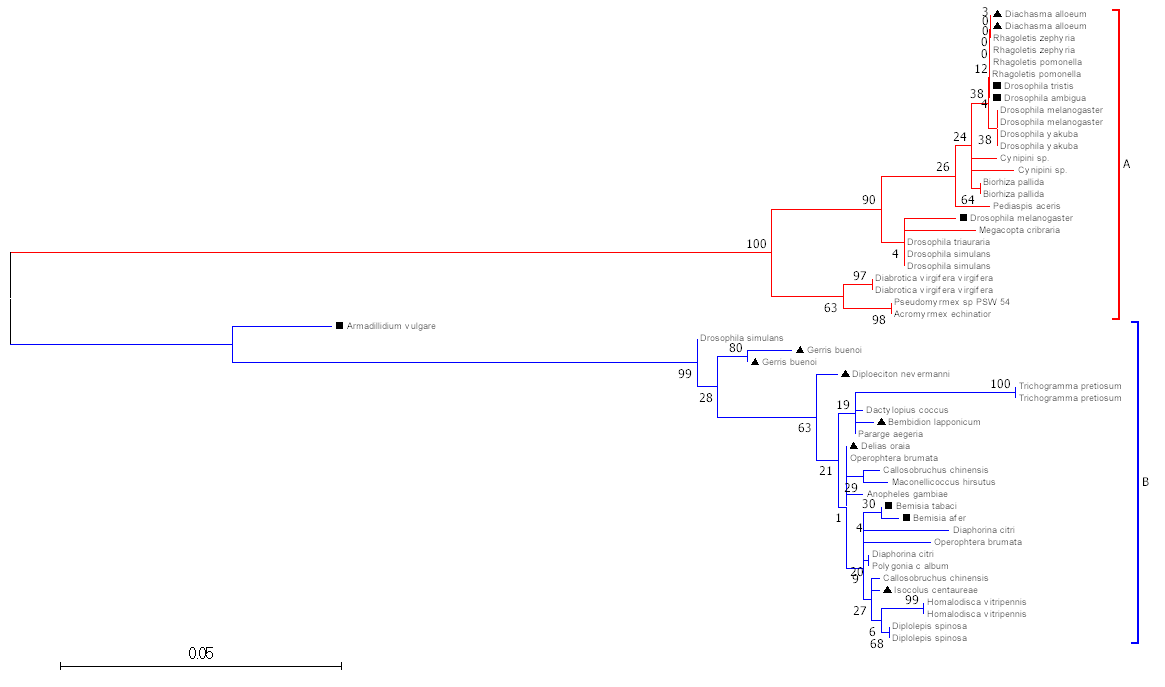
*
