# Supplementary figures and images for: A bioinformatics approach to identifying Wolbachia infections in arthropods
Source: PeerJ. 2018 Sep 3;6:e5486. doi: 10.7717/peerj.5486 (PMC6126470; doi:10.7717/peerj.5486)

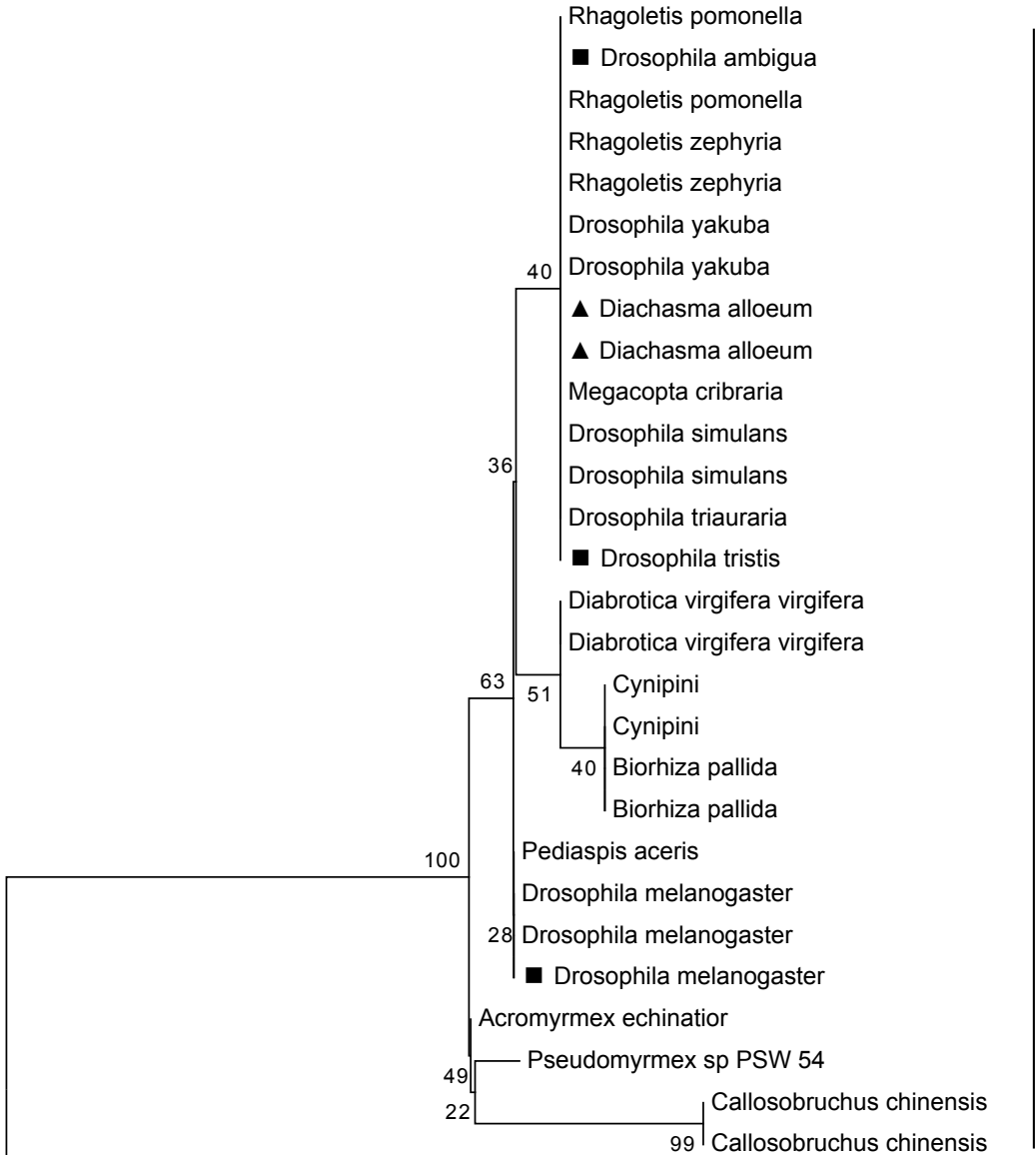

A

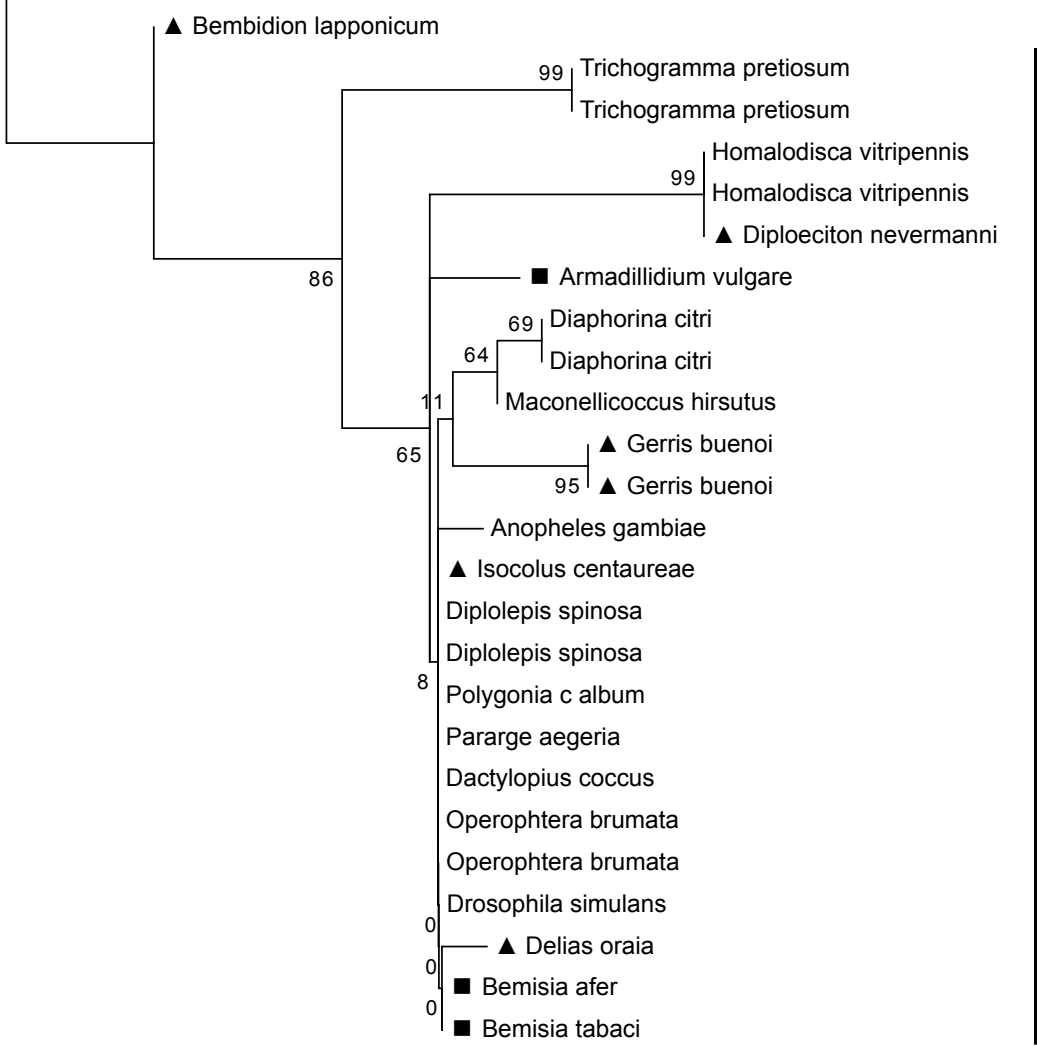

B

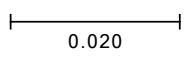

Supplement: Supplemental Information 1 — Supplementary figures/tables and code used for data analysis [file peerj-06-5486-s001.zip › Supplement/Phylogeny/concatenated_ftsZ_groE_phylogeny.pdf]

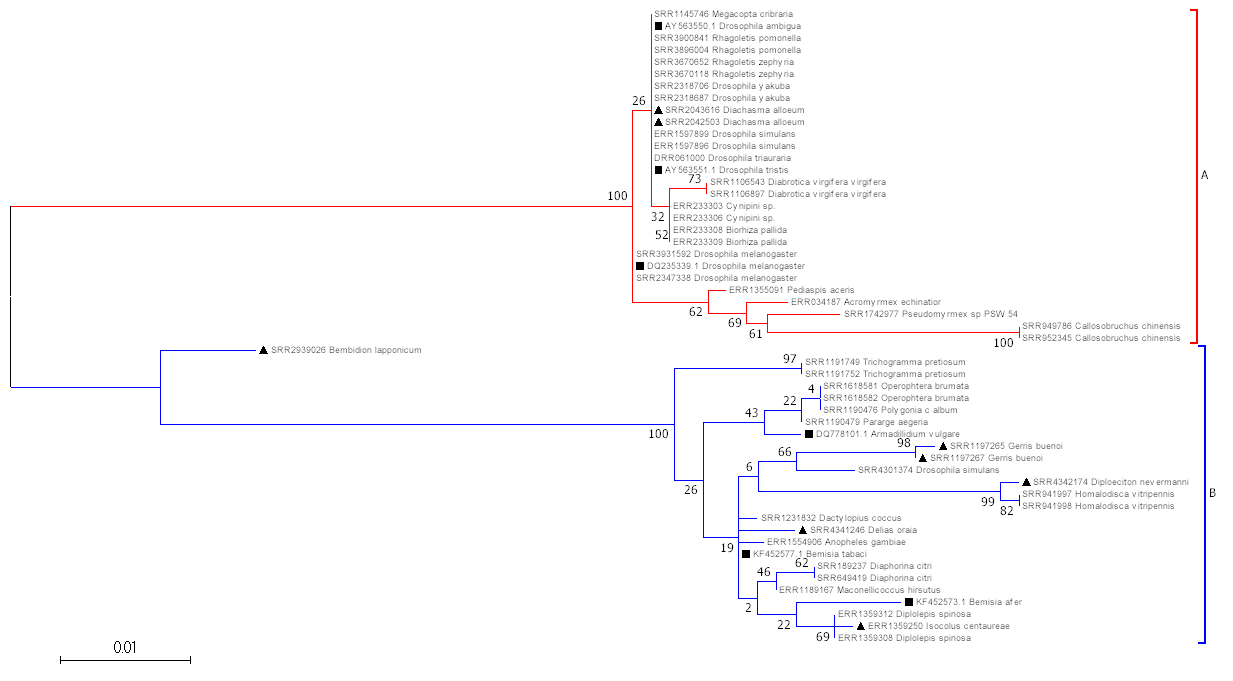

Supplement: Supplemental Information 1 — Supplementary figures/tables and code used for data analysis [file peerj-06-5486-s001.zip › Supplement/Phylogeny/ftsZ_phylogeny.png]

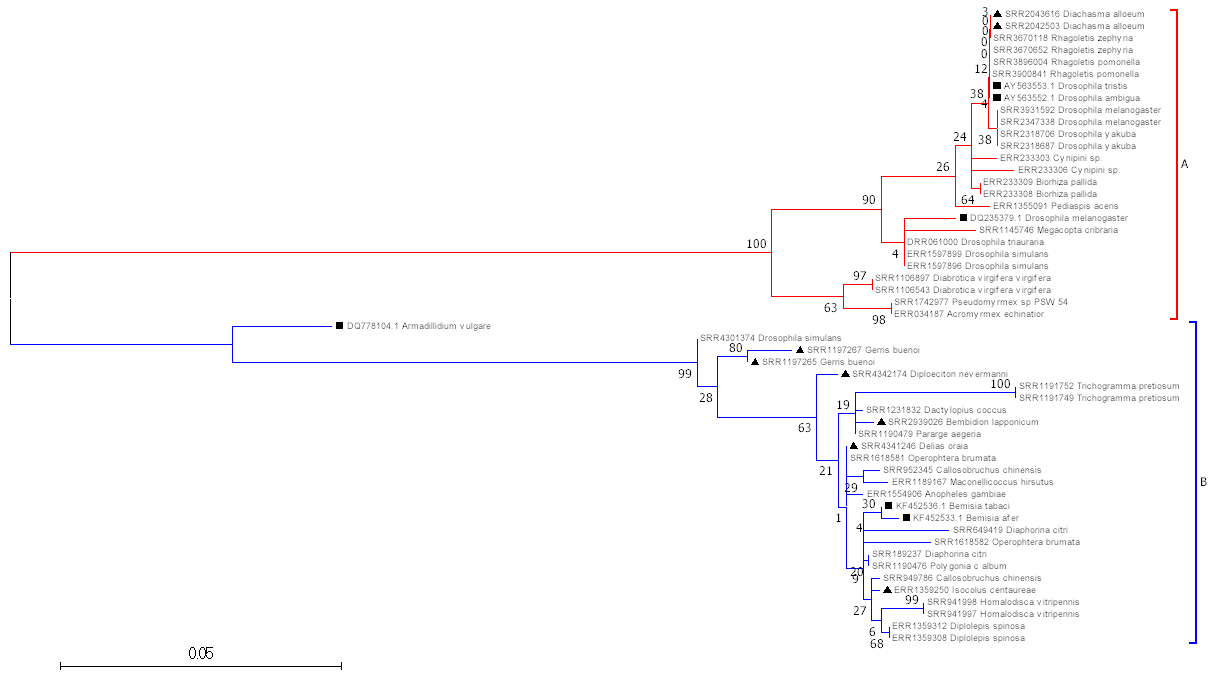

Supplement: Supplemental Information 1 — Supplementary figures/tables and code used for data analysis [file peerj-06-5486-s001.zip › Supplement/Phylogeny/groE_phylogeny.png]

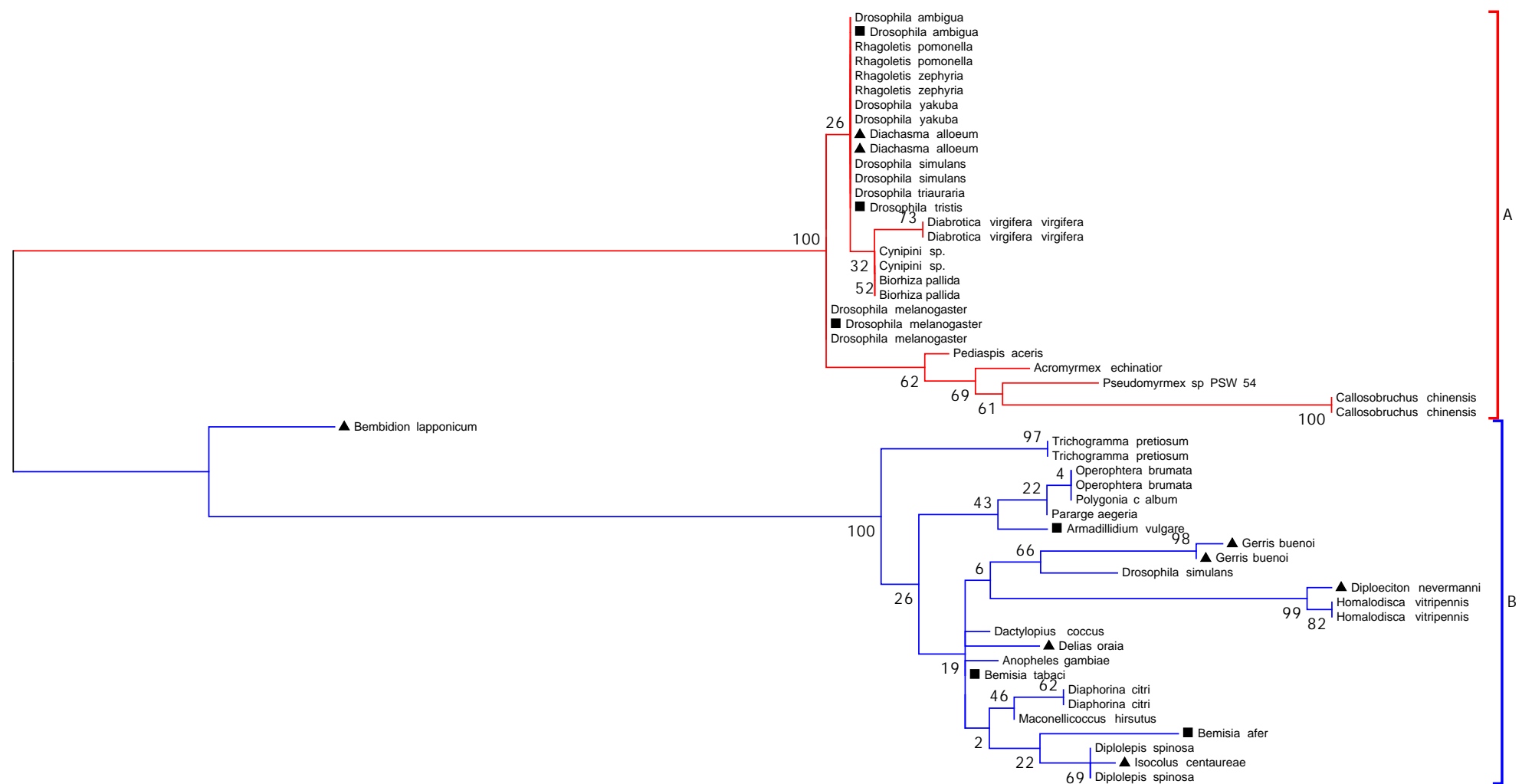

Supplement: Supplemental Information 1 — Supplementary figures/tables and code used for data analysis [file peerj-06-5486-s001.zip › Supplement/Supplementary Figure S1.pdf]

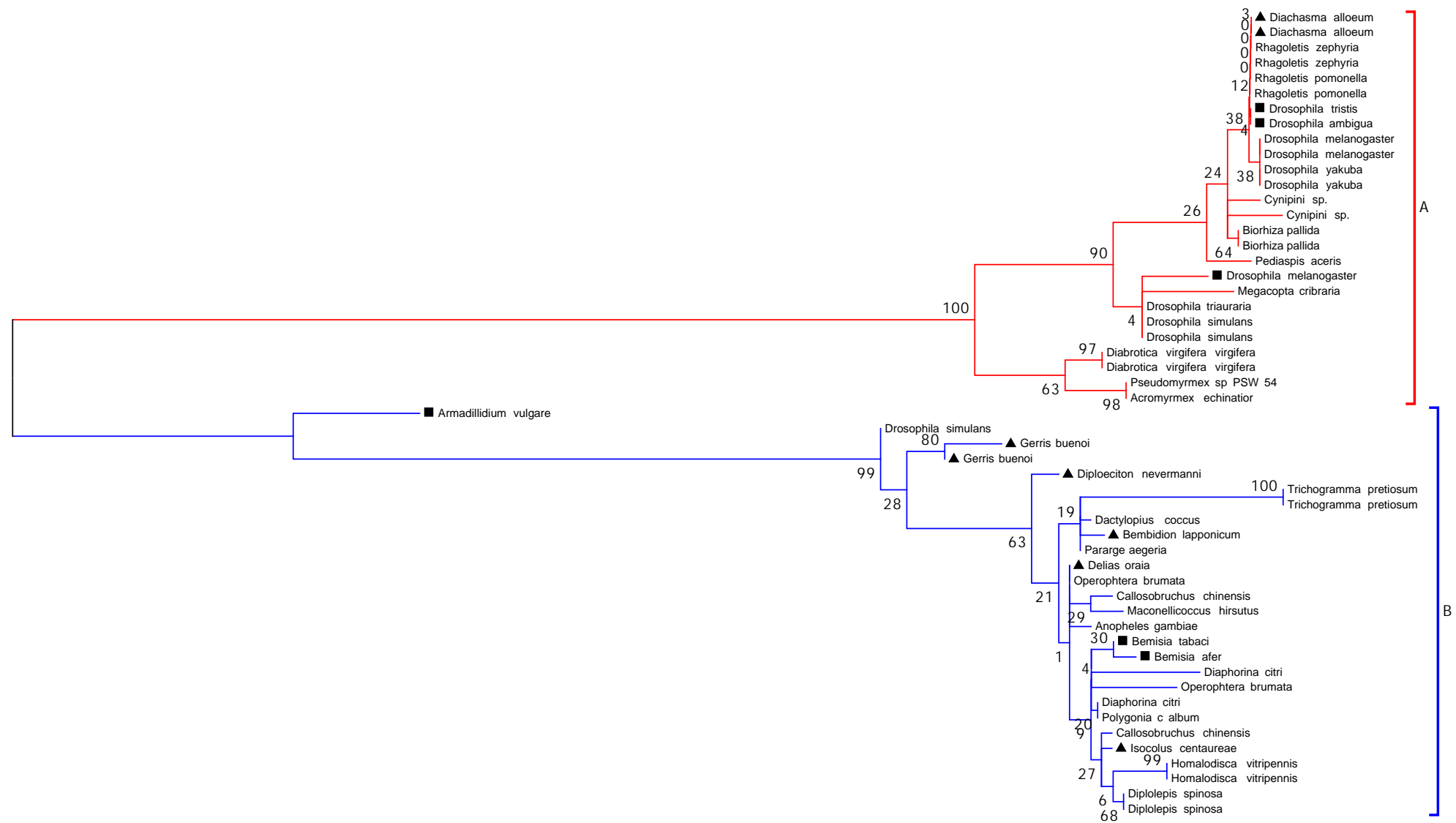

0.05

Supplement: Supplemental Information 1 — Supplementary figures/tables and code used for data analysis [file peerj-06-5486-s001.zip › Supplement/Supplementary Figure S2.pdf]

Total span assessed for coverage = 1.251 Mb  
Median coverage = 56.15

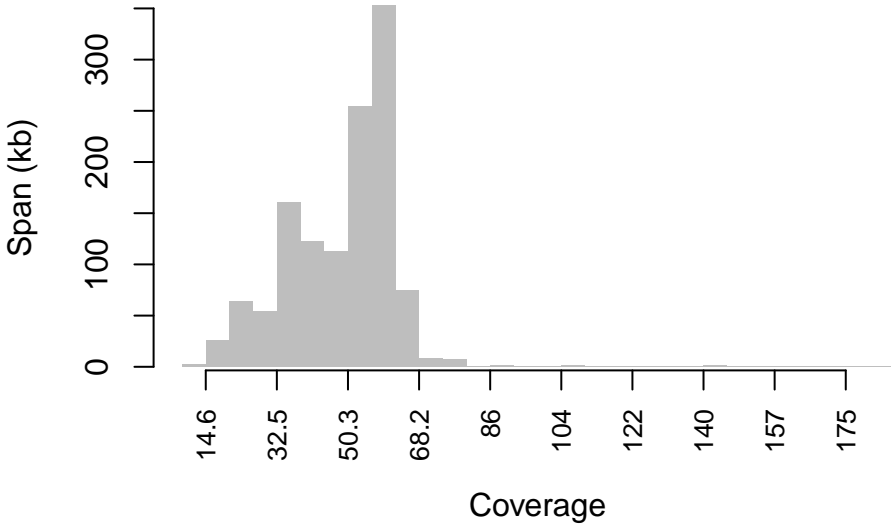

Supplement: Supplemental Information 1 — Supplementary figures/tables and code used for data analysis [file peerj-06-5486-s001.zip › Supplement/wolbachia_coverage_mapping/pdfs/Acromyrmex_echinatior.pdf]

Total span assessed for coverage = 1.187 Mb  
Median coverage = 8.95

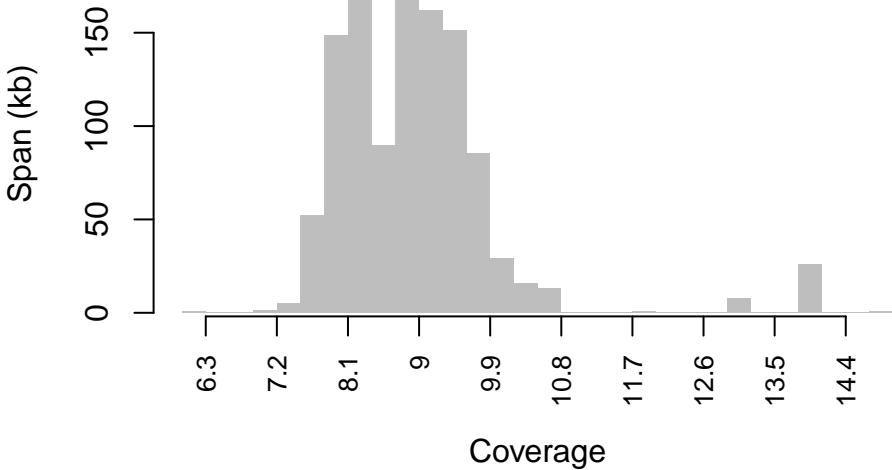

Supplement: Supplemental Information 1 — Supplementary figures/tables and code used for data analysis [file peerj-06-5486-s001.zip › Supplement/wolbachia_coverage_mapping/pdfs/Anopheles_gambiae.pdf]

Total span assessed for coverage = 0.817 Mb  
Median coverage = 10.68

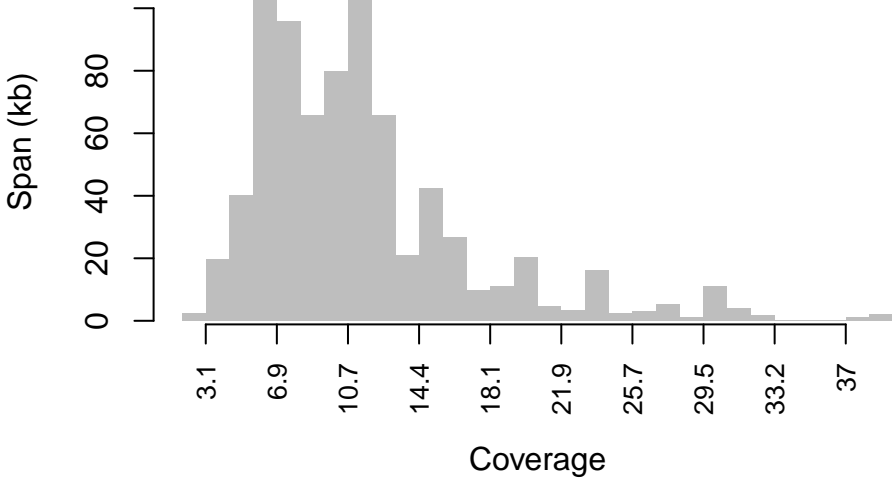

Supplement: Supplemental Information 1 — Supplementary figures/tables and code used for data analysis [file peerj-06-5486-s001.zip › Supplement/wolbachia_coverage_mapping/pdfs/Bembidion_lapponicum.pdf]

Total span assessed for coverage = 1.153 Mb  
Median coverage = 16.19

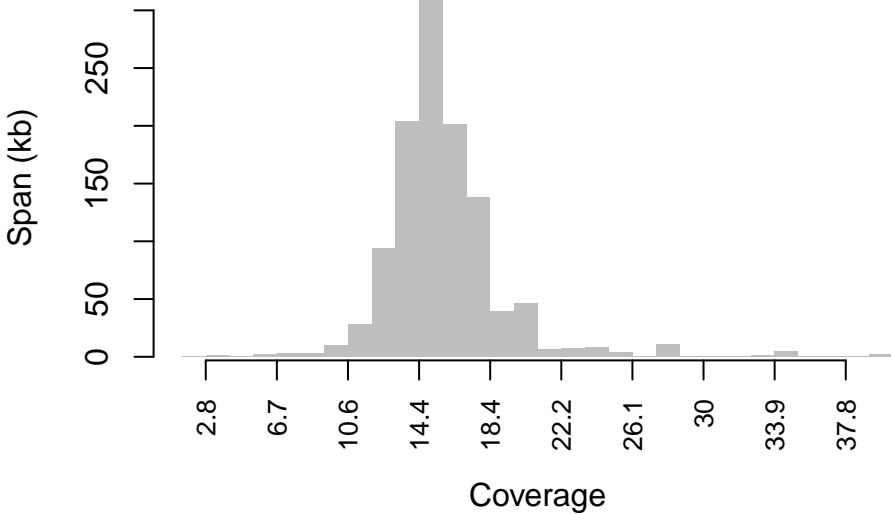

Supplement: Supplemental Information 1 — Supplementary figures/tables and code used for data analysis [file peerj-06-5486-s001.zip › Supplement/wolbachia_coverage_mapping/pdfs/Biorhiza_pallida_1.pdf]

Total span assessed for coverage = 1.147 Mb  
Median coverage = 15.78

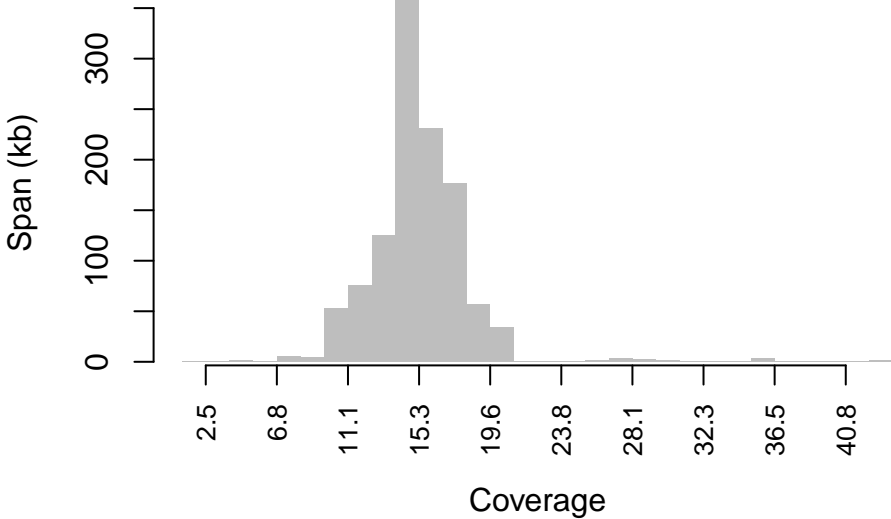

Supplement: Supplemental Information 1 — Supplementary figures/tables and code used for data analysis [file peerj-06-5486-s001.zip › Supplement/wolbachia_coverage_mapping/pdfs/Biorhiza_pallida_2.pdf]

Total span assessed for coverage = 0.189 Mb  
Median coverage = 4.19

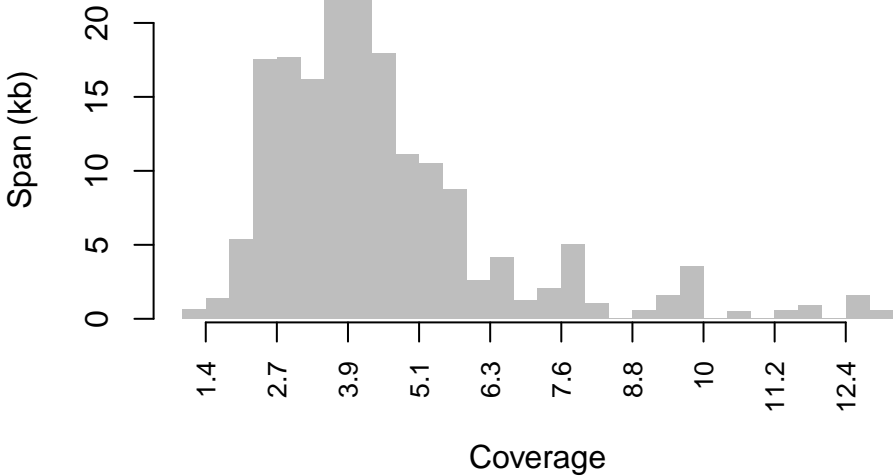

Supplement: Supplemental Information 1 — Supplementary figures/tables and code used for data analysis [file peerj-06-5486-s001.zip › Supplement/wolbachia_coverage_mapping/pdfs/Biorhiza_pallida_3.pdf]

Total span assessed for coverage = 2.238 Mb  
Median coverage = 335.15

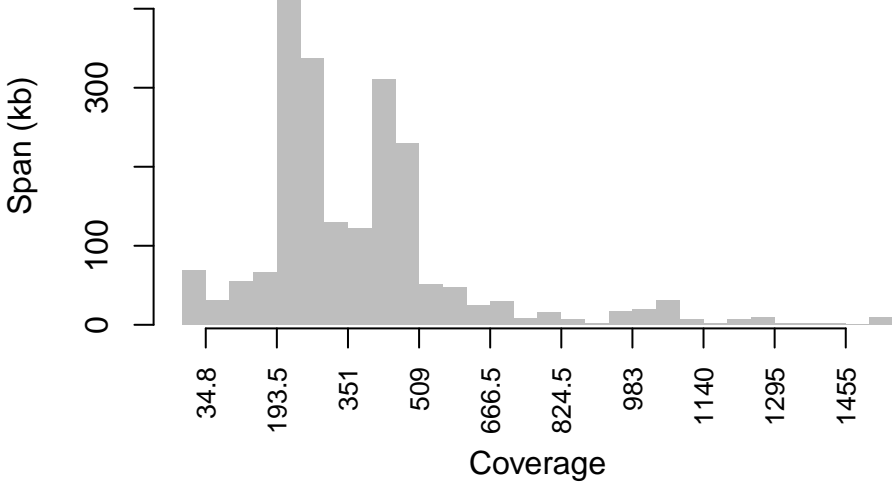

Supplement: Supplemental Information 1 — Supplementary figures/tables and code used for data analysis [file peerj-06-5486-s001.zip › Supplement/wolbachia_coverage_mapping/pdfs/Callosobruchus_chinensis.pdf]

Total span assessed for coverage = 0.771 Mb  
Median coverage = 11.36

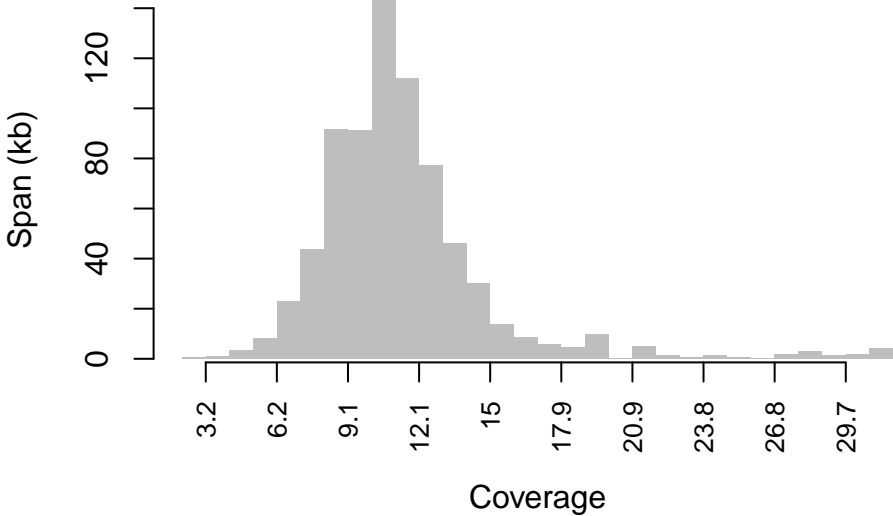

Supplement: Supplemental Information 1 — Supplementary figures/tables and code used for data analysis [file peerj-06-5486-s001.zip › Supplement/wolbachia_coverage_mapping/pdfs/Ceratina_calcarata_1.pdf]

Total span assessed for coverage = 1.058 Mb  
Median coverage = 19.56

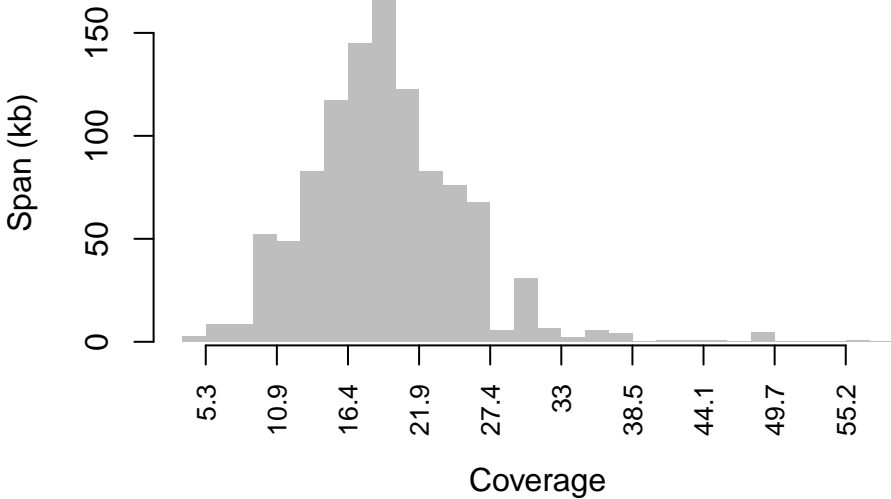

Supplement: Supplemental Information 1 — Supplementary figures/tables and code used for data analysis [file peerj-06-5486-s001.zip › Supplement/wolbachia_coverage_mapping/pdfs/Cynipini_1.pdf]

Total span assessed for coverage = 0.977 Mb  
Median coverage = 17.32

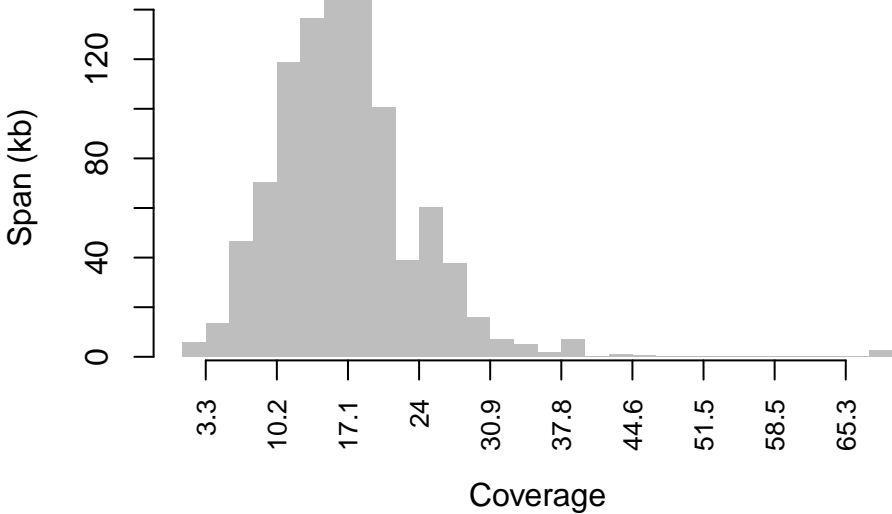

Supplement: Supplemental Information 1 — Supplementary figures/tables and code used for data analysis [file peerj-06-5486-s001.zip › Supplement/wolbachia_coverage_mapping/pdfs/Cynipini_2.pdf]

Total span assessed for coverage = 2.321 Mb  
Median coverage = 110.37

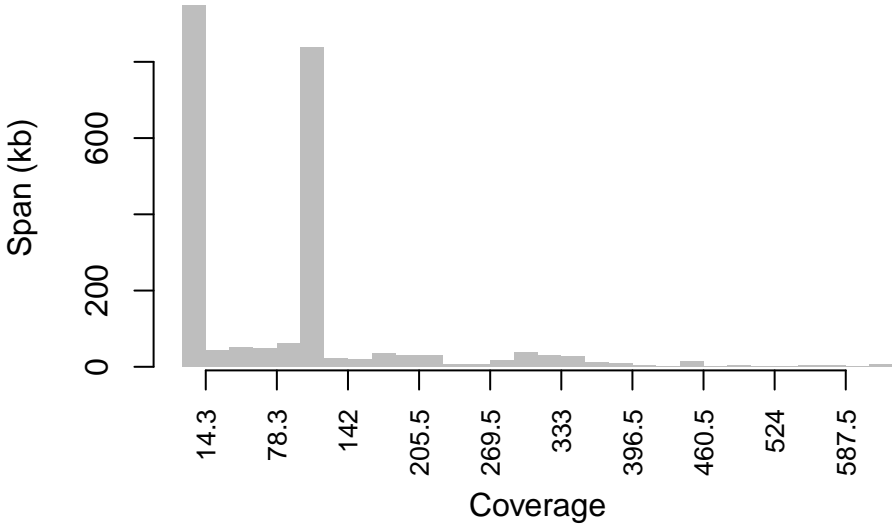

Supplement: Supplemental Information 1 — Supplementary figures/tables and code used for data analysis [file peerj-06-5486-s001.zip › Supplement/wolbachia_coverage_mapping/pdfs/Dactylopius_coccus.pdf]

Total span assessed for coverage = 1.143 Mb  
Median coverage = 26.35

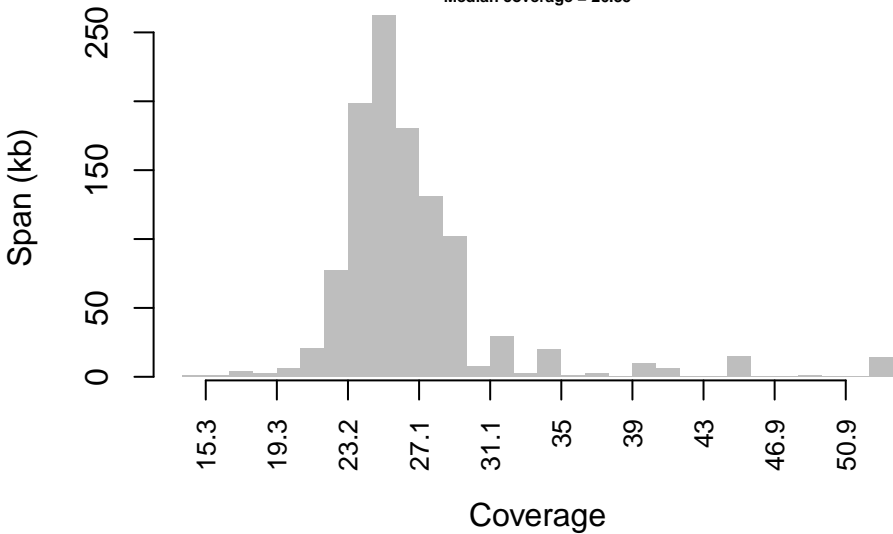

Supplement: Supplemental Information 1 — Supplementary figures/tables and code used for data analysis [file peerj-06-5486-s001.zip › Supplement/wolbachia_coverage_mapping/pdfs/Delias_oraia.pdf]

Total span assessed for coverage = 1.414 Mb  
Median coverage = 898.46

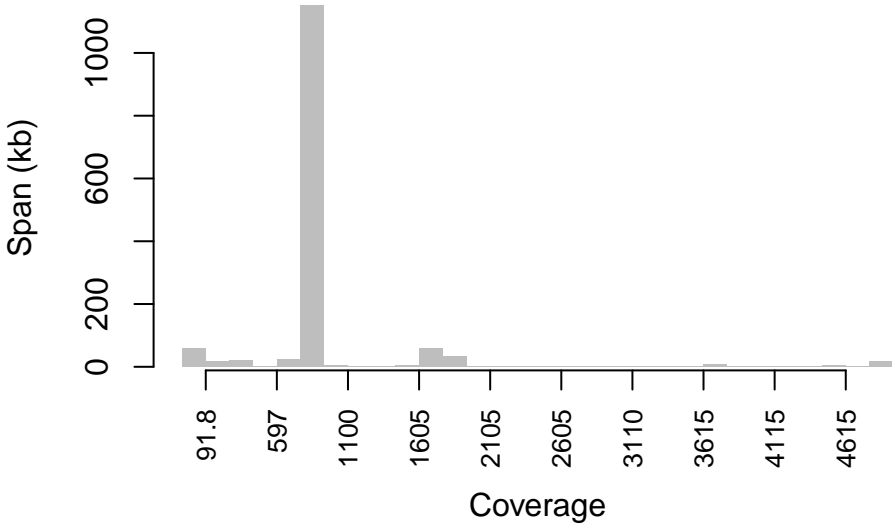

Supplement: Supplemental Information 1 — Supplementary figures/tables and code used for data analysis [file peerj-06-5486-s001.zip › Supplement/wolbachia_coverage_mapping/pdfs/Diabrotica_virgifera_virgifera_1.pdf]

Total span assessed for coverage = 1.398 Mb  
Median coverage = 753.69

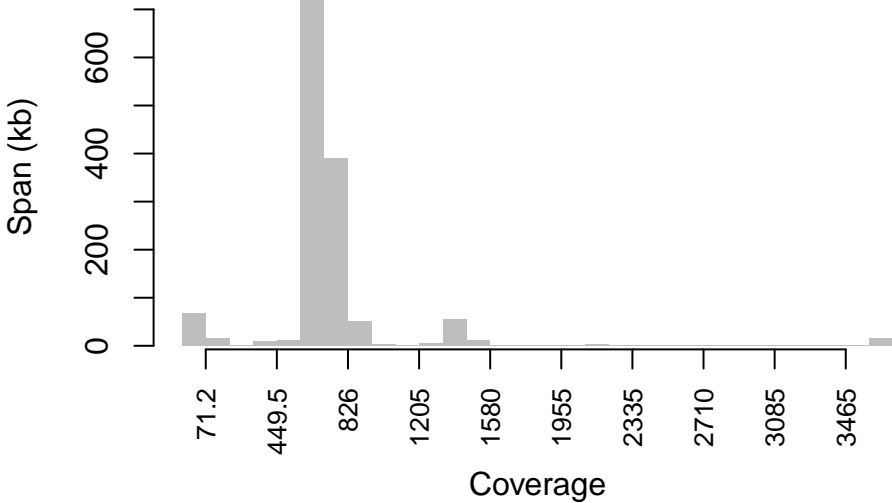

Supplement: Supplemental Information 1 — Supplementary figures/tables and code used for data analysis [file peerj-06-5486-s001.zip › Supplement/wolbachia_coverage_mapping/pdfs/Diabrotica_virgifera_virgifera_2.pdf]

Total span assessed for coverage = 1.318 Mb  
Median coverage = 655.74

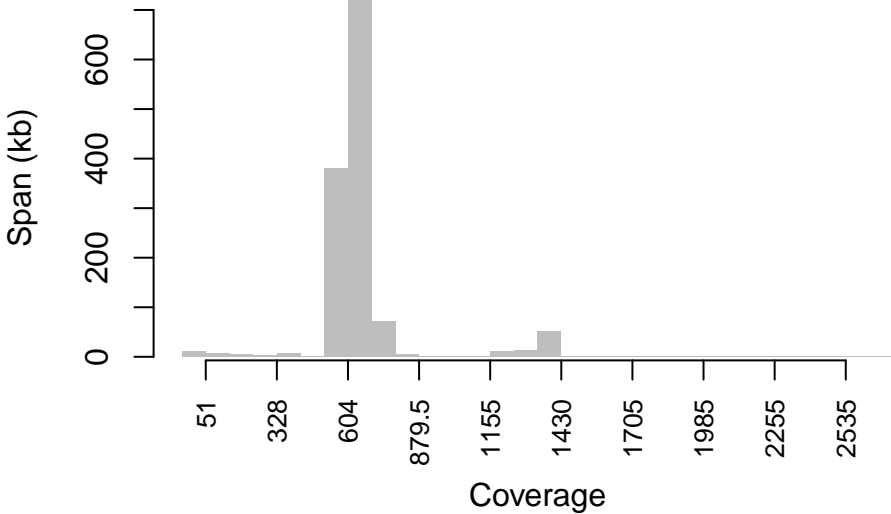

Supplement: Supplemental Information 1 — Supplementary figures/tables and code used for data analysis [file peerj-06-5486-s001.zip › Supplement/wolbachia_coverage_mapping/pdfs/Diabrotica_virgifera_virgifera_3.pdf]

Total span assessed for coverage = 1.294 Mb  
Median coverage = 825.73

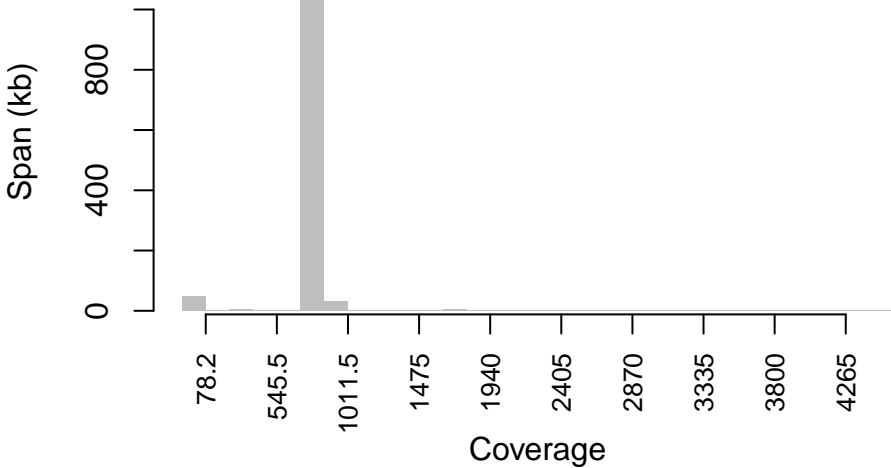

Supplement: Supplemental Information 1 — Supplementary figures/tables and code used for data analysis [file peerj-06-5486-s001.zip › Supplement/wolbachia_coverage_mapping/pdfs/Diachasma_alloeum.pdf]

Total span assessed for coverage = 1.302 Mb  
Median coverage = 177.89

Span (kb)

600  
200  
0

77.3

206.5

334.5

463.5

591.5

720.5

848.5

977

1105

1235

Coverage

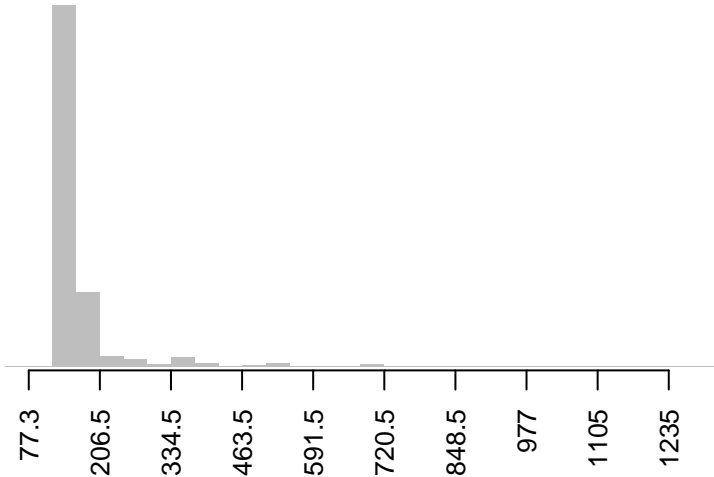

Supplement: Supplemental Information 1 — Supplementary figures/tables and code used for data analysis [file peerj-06-5486-s001.zip › Supplement/wolbachia_coverage_mapping/pdfs/Diaphorina_citri_1.pdf]

Total span assessed for coverage = 1.348 Mb  
Median coverage = 253.94

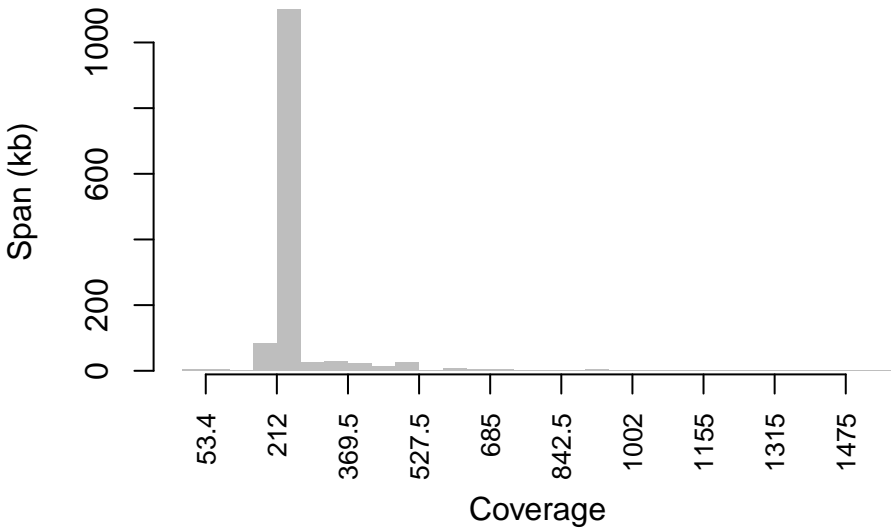

Supplement: Supplemental Information 1 — Supplementary figures/tables and code used for data analysis [file peerj-06-5486-s001.zip › Supplement/wolbachia_coverage_mapping/pdfs/Diaphorina_citri_2.pdf]

Total span assessed for coverage = 1.528 Mb  
Median coverage = 62.89

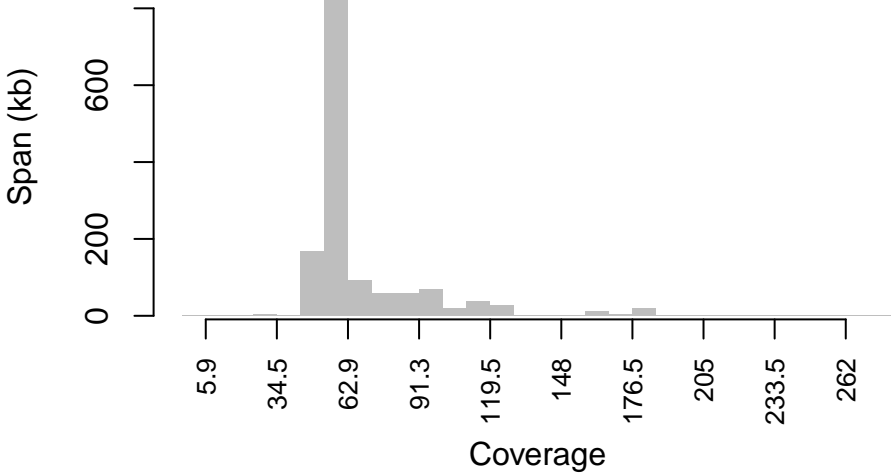

Supplement: Supplemental Information 1 — Supplementary figures/tables and code used for data analysis [file peerj-06-5486-s001.zip › Supplement/wolbachia_coverage_mapping/pdfs/Diploeciton_nevermanni.pdf]

Span (kb)

400  
300  
200  
100  
0

6.9

18.9

30.8

42.6

54.5

66.4

78.2

90.2

102

114

Coverage

Total span assessed for coverage = 1.315 Mb  
Median coverage = 57.45

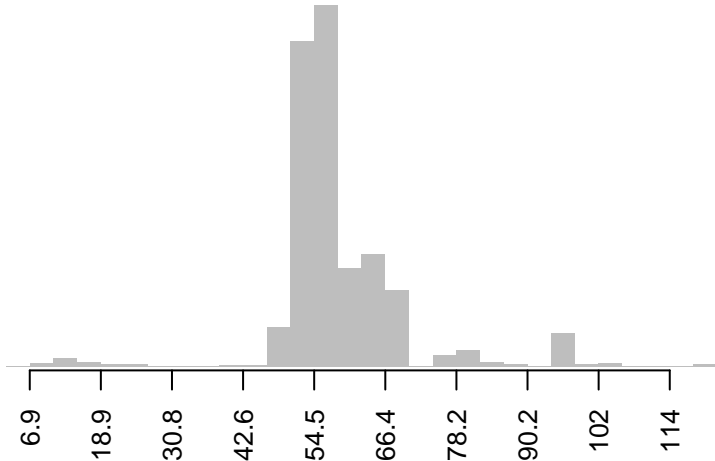

Supplement: Supplemental Information 1 — Supplementary figures/tables and code used for data analysis [file peerj-06-5486-s001.zip › Supplement/wolbachia_coverage_mapping/pdfs/Diplolepis_spinosa_1.pdf]

Total span assessed for coverage = 1.261 Mb  
Median coverage = 53.32

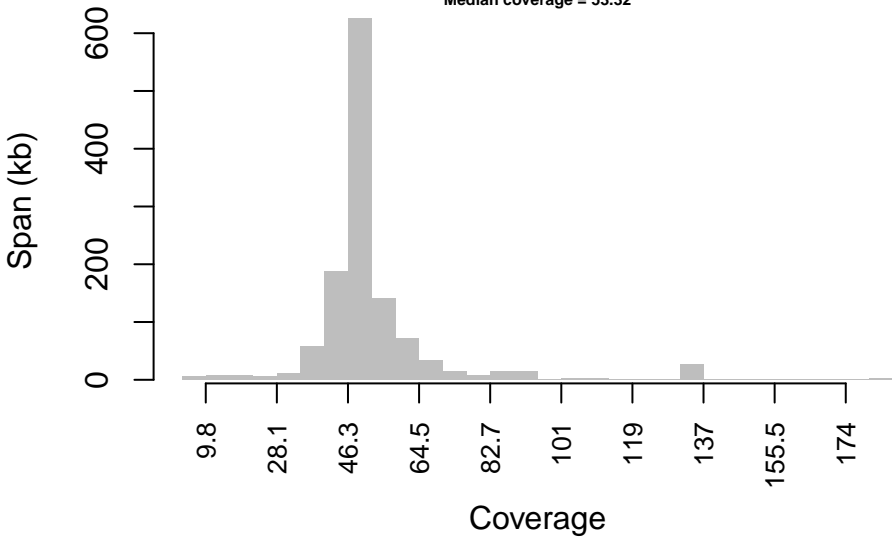

Supplement: Supplemental Information 1 — Supplementary figures/tables and code used for data analysis [file peerj-06-5486-s001.zip › Supplement/wolbachia_coverage_mapping/pdfs/Diplolepis_spinosa_2.pdf]

Total span assessed for coverage = 1.159 Mb  
Median coverage = 1617.49

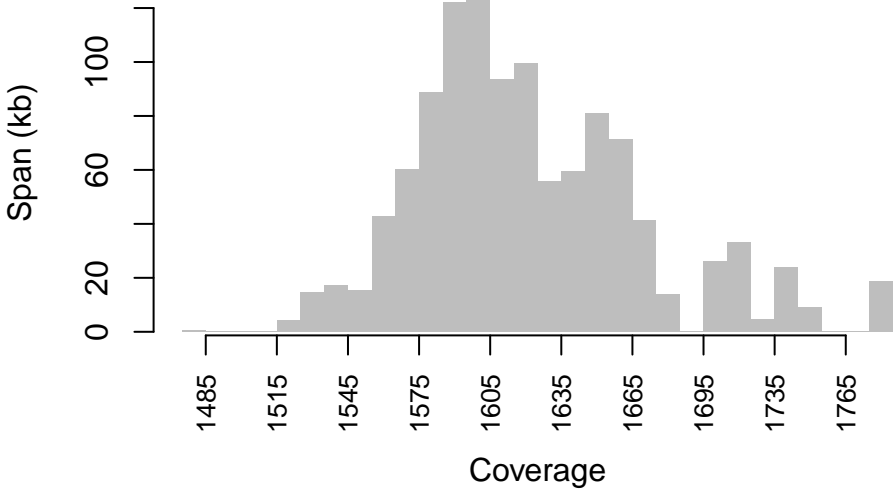

Supplement: Supplemental Information 1 — Supplementary figures/tables and code used for data analysis [file peerj-06-5486-s001.zip › Supplement/wolbachia_coverage_mapping/pdfs/Drosophila_melanogaster_1.pdf]

Total span assessed for coverage = 1.136 Mb  
Median coverage = 21.21

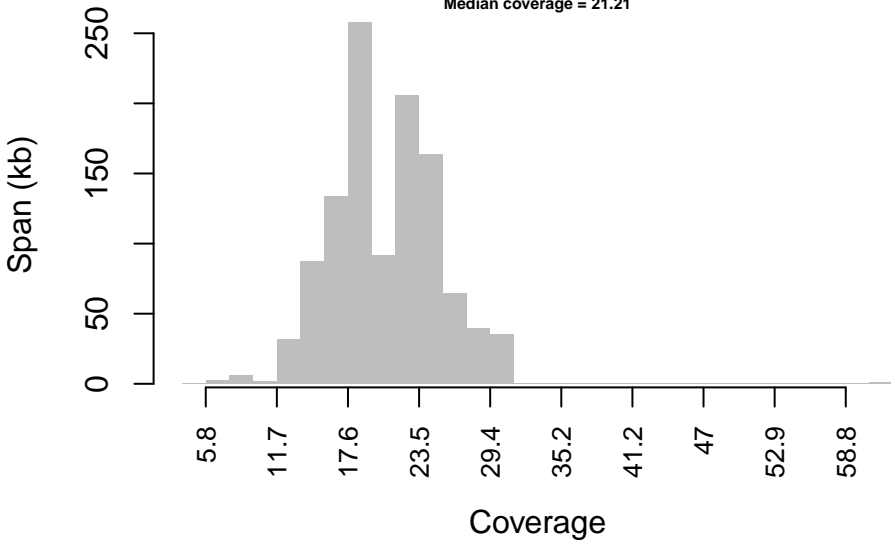

Supplement: Supplemental Information 1 — Supplementary figures/tables and code used for data analysis [file peerj-06-5486-s001.zip › Supplement/wolbachia_coverage_mapping/pdfs/Drosophila_melanogaster_2.pdf]

Total span assessed for coverage = 1.040 Mb  
Median coverage = 6.49

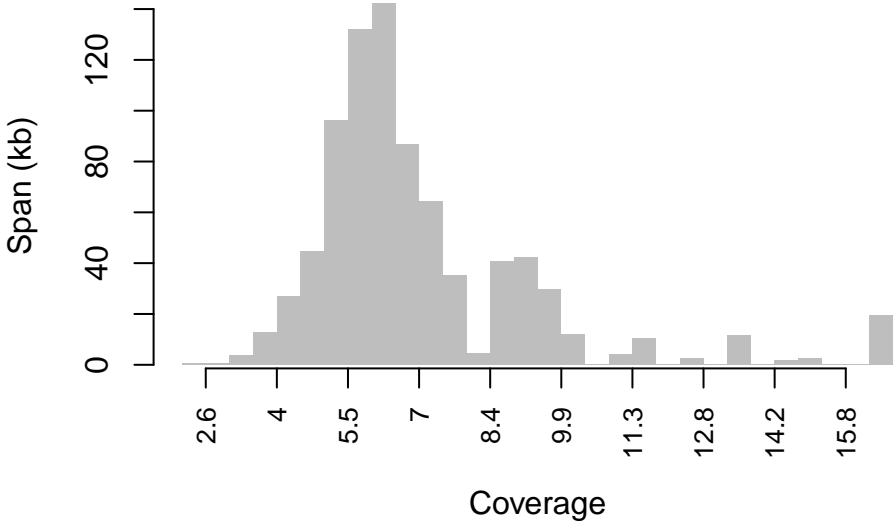

Supplement: Supplemental Information 1 — Supplementary figures/tables and code used for data analysis [file peerj-06-5486-s001.zip › Supplement/wolbachia_coverage_mapping/pdfs/Drosophila_melanogaster_3.pdf]

Total span assessed for coverage = 1.204 Mb  
Median coverage = 385.47

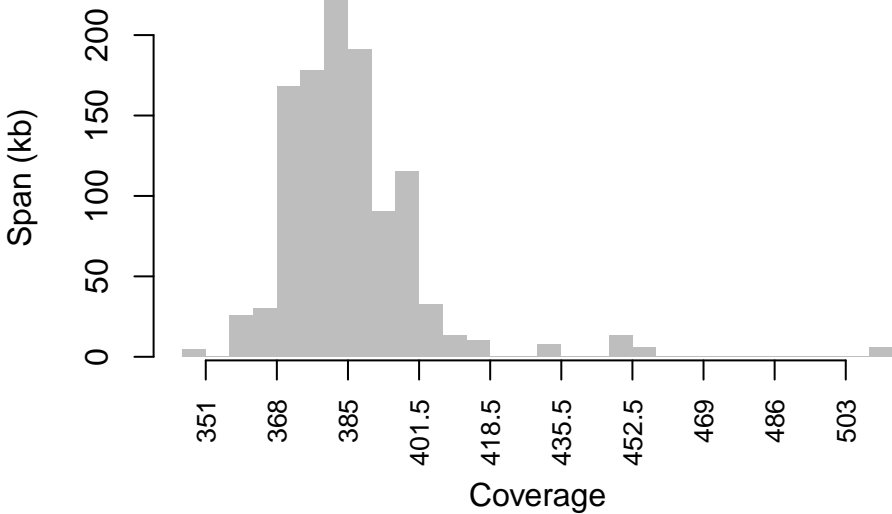

Supplement: Supplemental Information 1 — Supplementary figures/tables and code used for data analysis [file peerj-06-5486-s001.zip › Supplement/wolbachia_coverage_mapping/pdfs/Drosophila_simulans_1.pdf]

Total span assessed for coverage = 1.205 Mb  
Median coverage = 1070.50

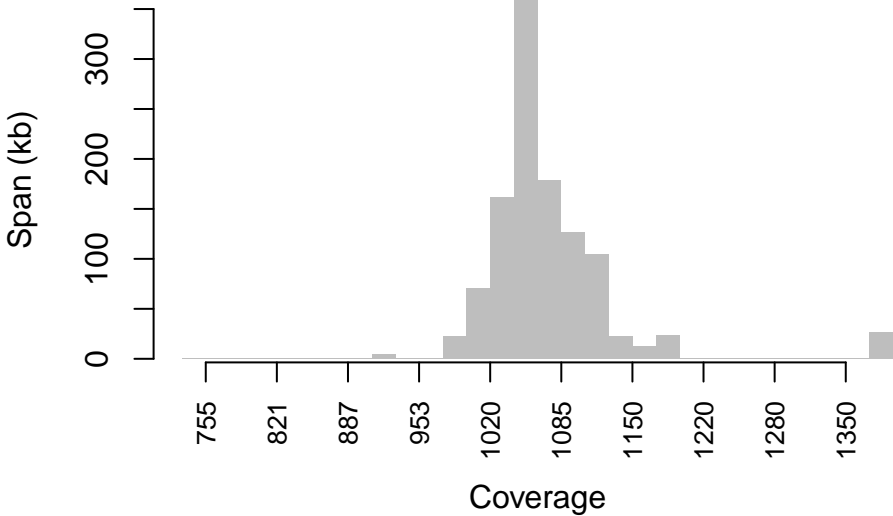

Supplement: Supplemental Information 1 — Supplementary figures/tables and code used for data analysis [file peerj-06-5486-s001.zip › Supplement/wolbachia_coverage_mapping/pdfs/Drosophila_simulans_2.pdf]

Total span assessed for coverage = 1.212 Mb  
Median coverage = 1591.47

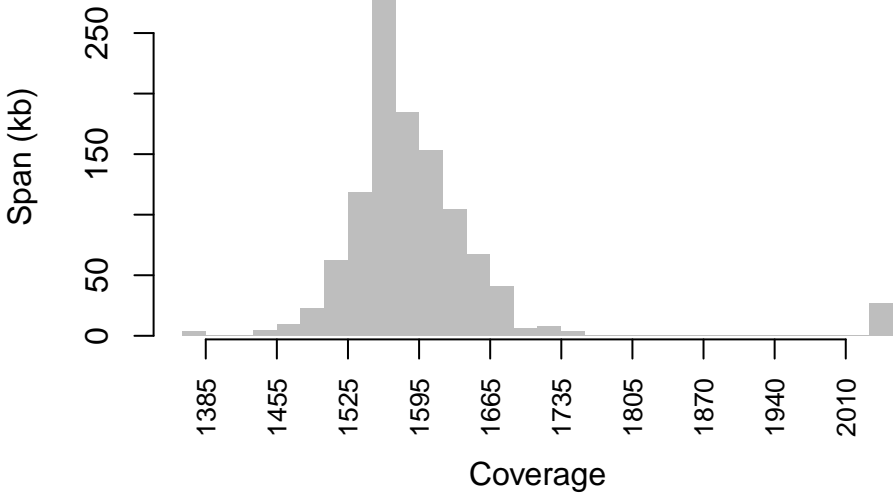

Supplement: Supplemental Information 1 — Supplementary figures/tables and code used for data analysis [file peerj-06-5486-s001.zip › Supplement/wolbachia_coverage_mapping/pdfs/Drosophila_simulans_3.pdf]

Total span assessed for coverage = 1.254 Mb  
Median coverage = 153.65

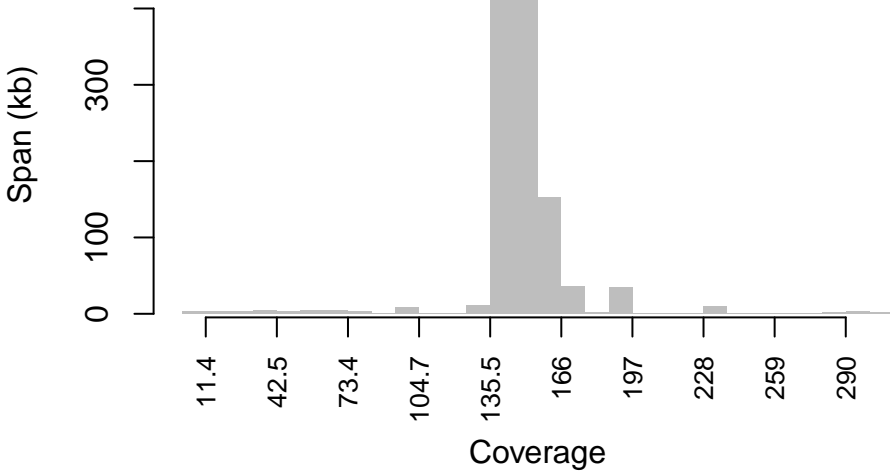

Supplement: Supplemental Information 1 — Supplementary figures/tables and code used for data analysis [file peerj-06-5486-s001.zip › Supplement/wolbachia_coverage_mapping/pdfs/Drosophila_triauraria_1.pdf]

Total span assessed for coverage = 1.177 Mb  
Median coverage = 42.10

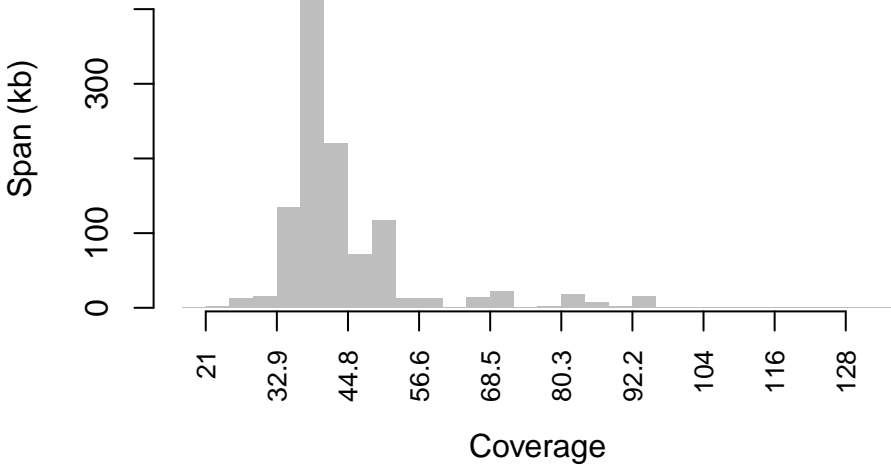

Supplement: Supplemental Information 1 — Supplementary figures/tables and code used for data analysis [file peerj-06-5486-s001.zip › Supplement/wolbachia_coverage_mapping/pdfs/Drosophila_yakuba_1.pdf]

Total span assessed for coverage = 1.169 Mb  
Median coverage = 34.74

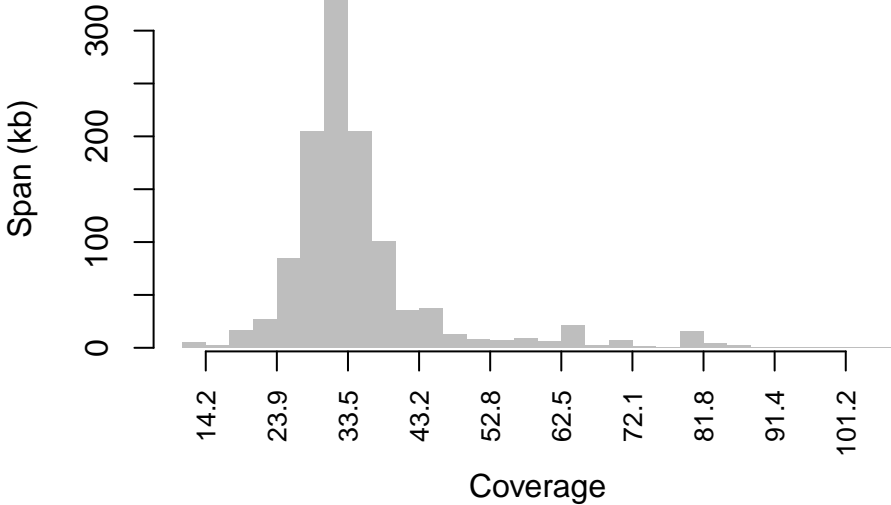

Supplement: Supplemental Information 1 — Supplementary figures/tables and code used for data analysis [file peerj-06-5486-s001.zip › Supplement/wolbachia_coverage_mapping/pdfs/Drosophila_yakuba_2.pdf]

Total span assessed for coverage = 1.378 Mb  
Median coverage = 166.27

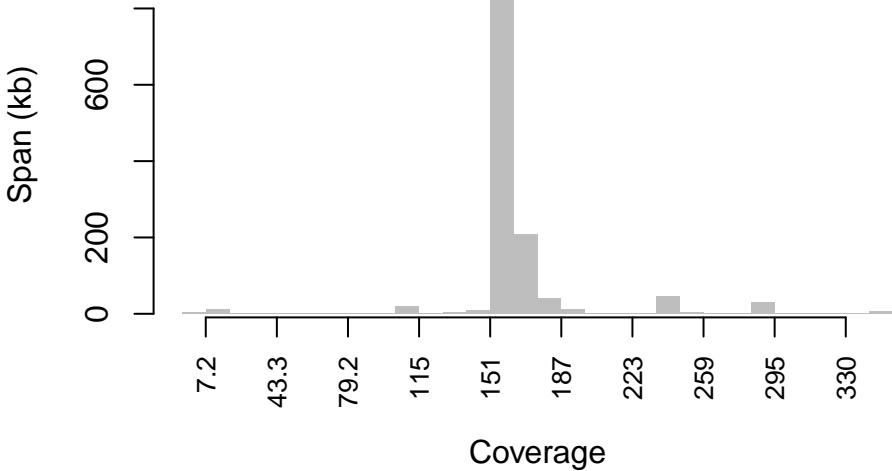

Supplement: Supplemental Information 1 — Supplementary figures/tables and code used for data analysis [file peerj-06-5486-s001.zip › Supplement/wolbachia_coverage_mapping/pdfs/Ecitophya_simulans.pdf]

Total span assessed for coverage = 1.377 Mb  
Median coverage = 69.40

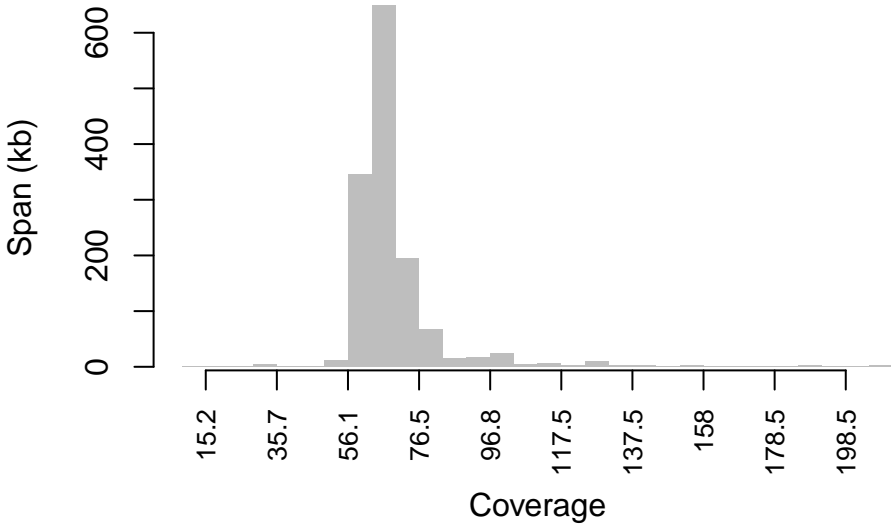

Supplement: Supplemental Information 1 — Supplementary figures/tables and code used for data analysis [file peerj-06-5486-s001.zip › Supplement/wolbachia_coverage_mapping/pdfs/Gerris_buenoi_1.pdf]

Total span assessed for coverage = 1.356 Mb  
Median coverage = 39.22

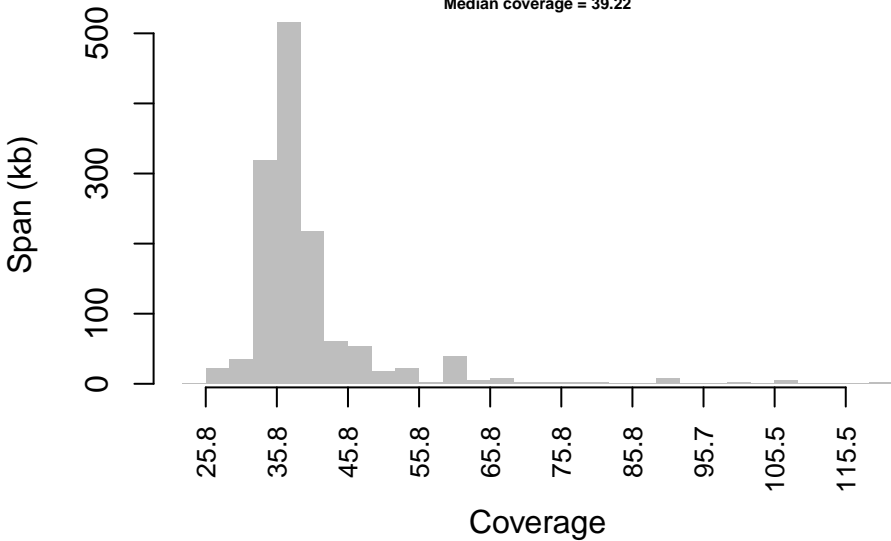

Supplement: Supplemental Information 1 — Supplementary figures/tables and code used for data analysis [file peerj-06-5486-s001.zip › Supplement/wolbachia_coverage_mapping/pdfs/Gerris_buenoi_2.pdf]

Total span assessed for coverage = 1.524 Mb  
Median coverage = 263.07

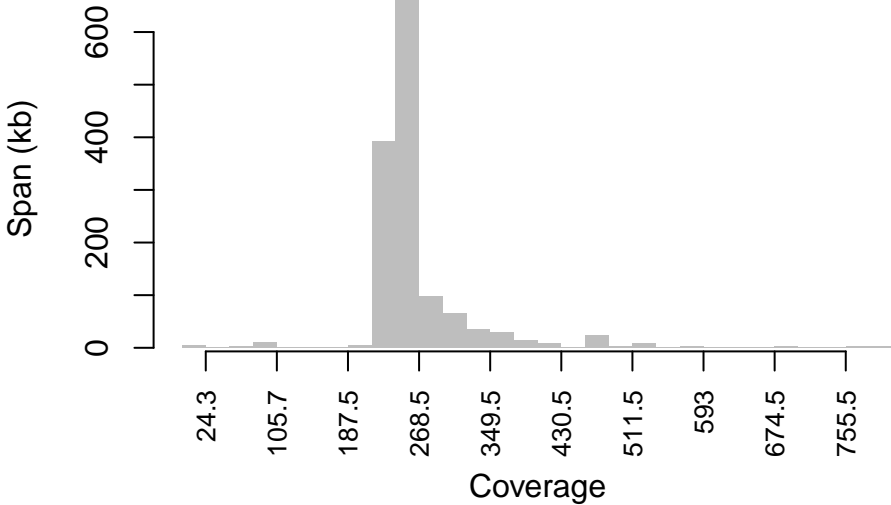

Supplement: Supplemental Information 1 — Supplementary figures/tables and code used for data analysis [file peerj-06-5486-s001.zip › Supplement/wolbachia_coverage_mapping/pdfs/Homalodisca_vitripennis_1.pdf]

Total span assessed for coverage = 1.634 Mb  
Median coverage = 64.15

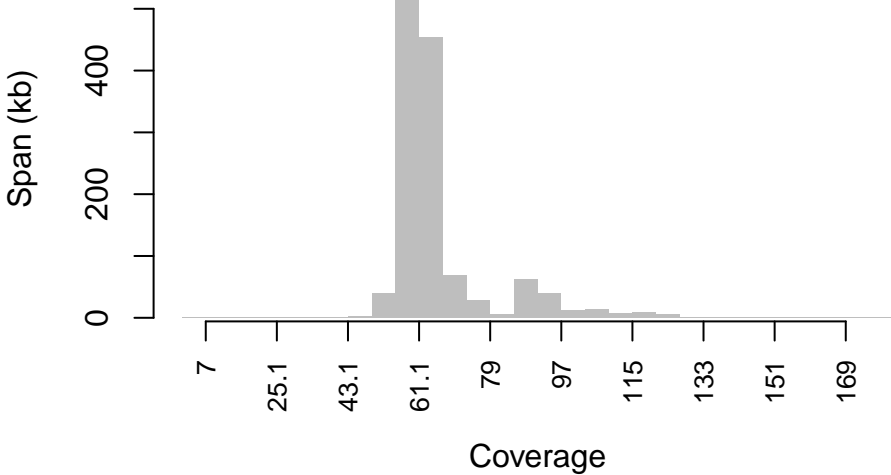

Supplement: Supplemental Information 1 — Supplementary figures/tables and code used for data analysis [file peerj-06-5486-s001.zip › Supplement/wolbachia_coverage_mapping/pdfs/Homalodisca_vitripennis_2.pdf]

Total span assessed for coverage = 0.807 Mb  
Median coverage = 7.87

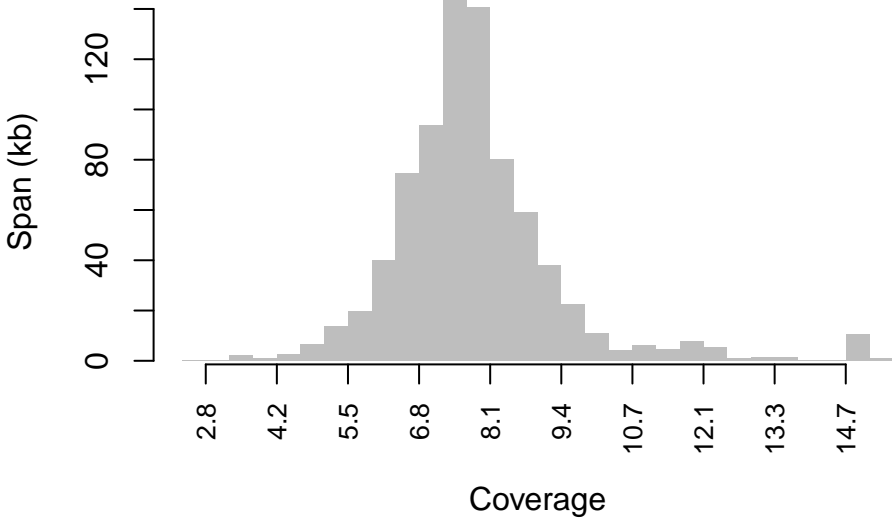

Supplement: Supplemental Information 1 — Supplementary figures/tables and code used for data analysis [file peerj-06-5486-s001.zip › Supplement/wolbachia_coverage_mapping/pdfs/Isocolus_centaureae_1.pdf]

Total span assessed for coverage = 0.767 Mb  
Median coverage = 7.72

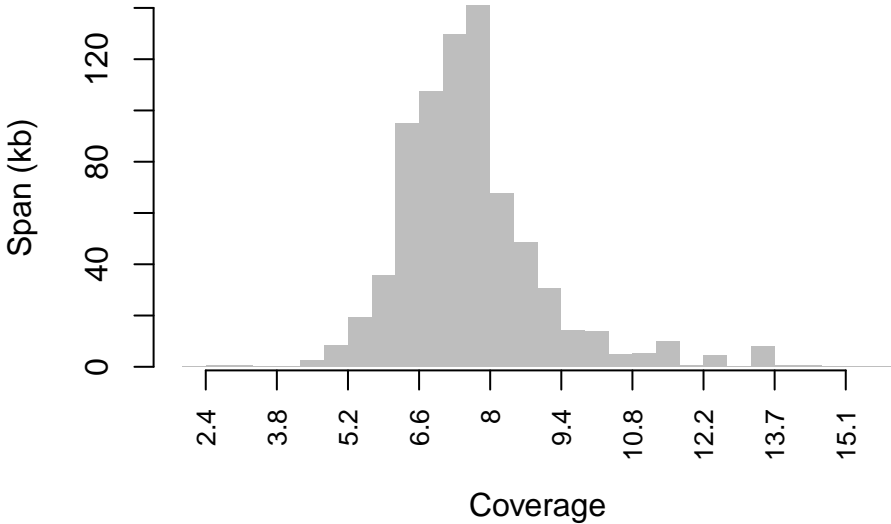

Supplement: Supplemental Information 1 — Supplementary figures/tables and code used for data analysis [file peerj-06-5486-s001.zip › Supplement/wolbachia_coverage_mapping/pdfs/Isocolus_centaureae_2.pdf]

Span (kb)

600  
400  
200  
0

5.1

28

50.8

73.6

96.3

119

142

164.5

187.5

210

Coverage

Total span assessed for coverage = 1.357 Mb  
Median coverage = 79.67

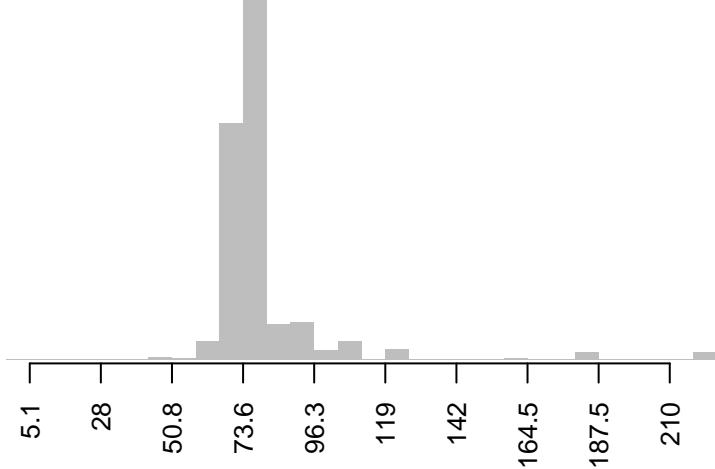

Supplement: Supplemental Information 1 — Supplementary figures/tables and code used for data analysis [file peerj-06-5486-s001.zip › Supplement/wolbachia_coverage_mapping/pdfs/Maconellicoccus_hirsutus.pdf]

Total span assessed for coverage = 1.207 Mb  
Median coverage = 31.11

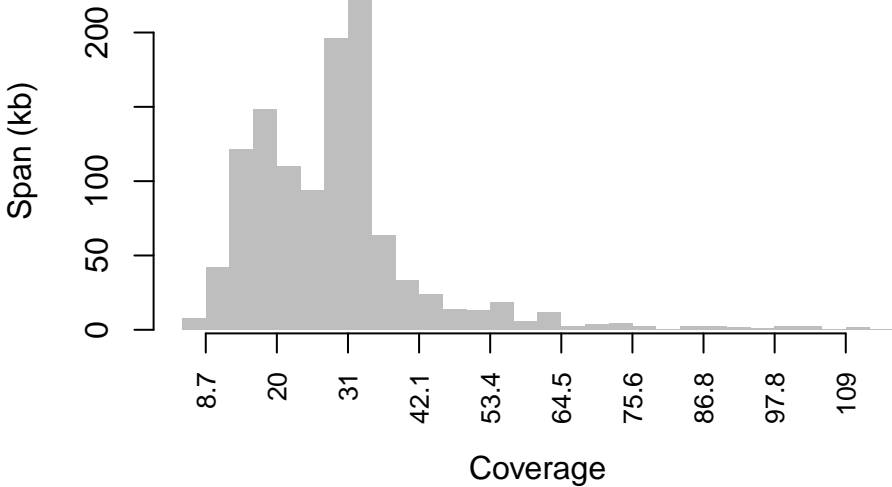

Supplement: Supplemental Information 1 — Supplementary figures/tables and code used for data analysis [file peerj-06-5486-s001.zip › Supplement/wolbachia_coverage_mapping/pdfs/Megacopta_cribraria.pdf]

Total span assessed for coverage = 0.963 Mb  
Median coverage = 9.91

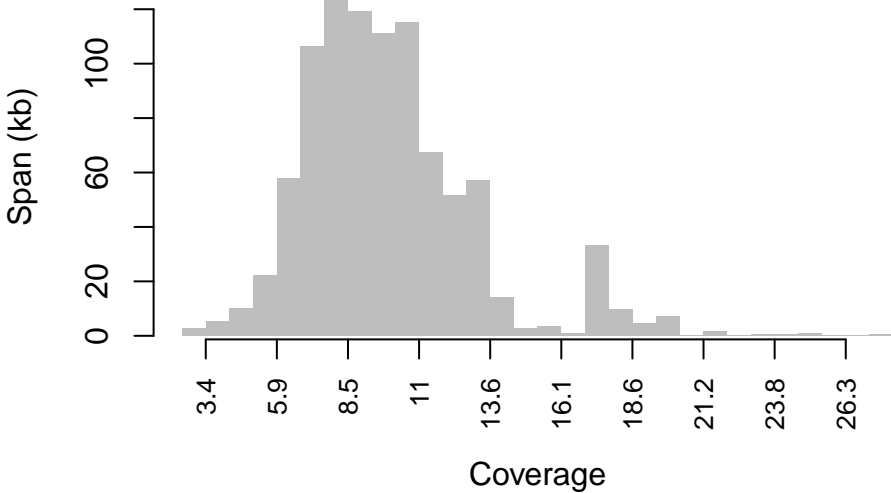

Supplement: Supplemental Information 1 — Supplementary figures/tables and code used for data analysis [file peerj-06-5486-s001.zip › Supplement/wolbachia_coverage_mapping/pdfs/Mycopsylla_fici_1.pdf]

Total span assessed for coverage = 1.044 Mb  
Median coverage = 11.82

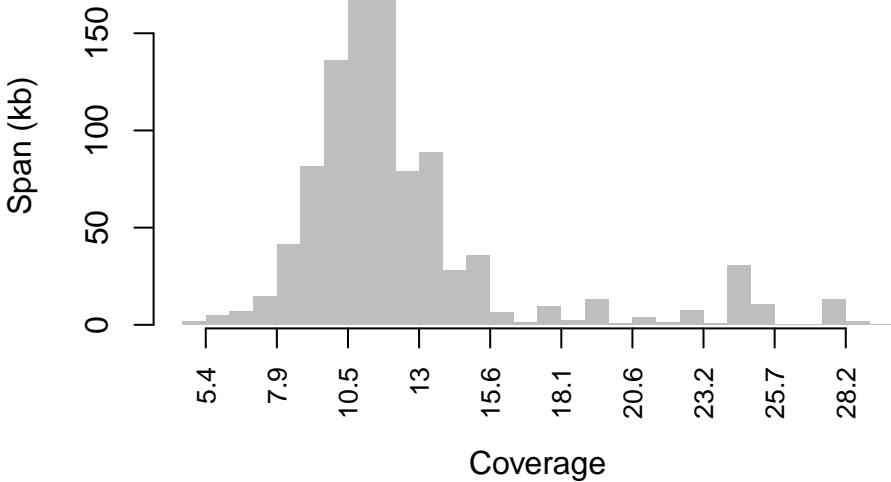

Supplement: Supplemental Information 1 — Supplementary figures/tables and code used for data analysis [file peerj-06-5486-s001.zip › Supplement/wolbachia_coverage_mapping/pdfs/Mycopsylla_fici_2.pdf]

Total span assessed for coverage = 0.213 Mb  
Median coverage = 5.67

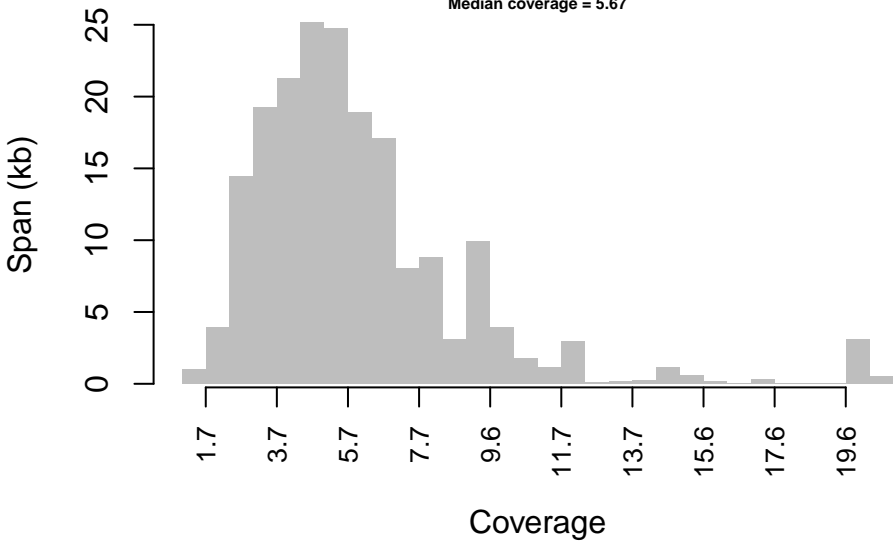

Supplement: Supplemental Information 1 — Supplementary figures/tables and code used for data analysis [file peerj-06-5486-s001.zip › Supplement/wolbachia_coverage_mapping/pdfs/Mycopsylla_proxima.pdf]

Total span assessed for coverage = 1.239 Mb  
Median coverage = 27.70

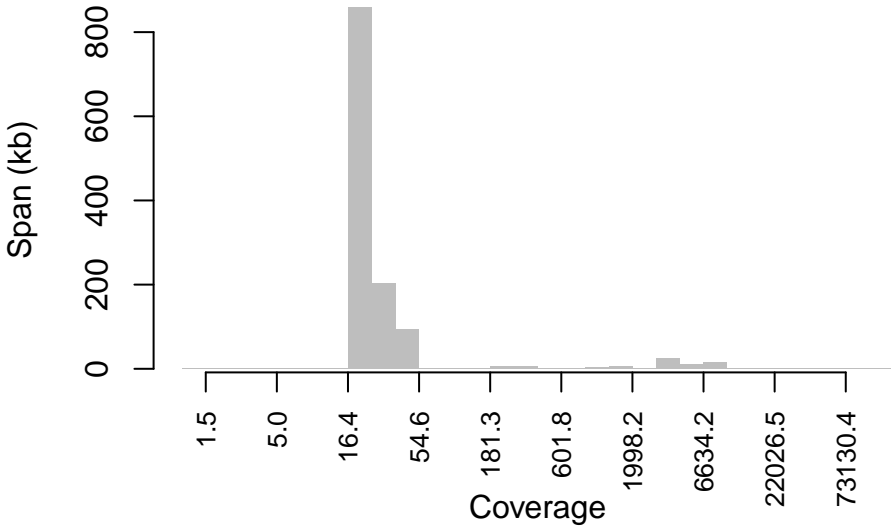

Supplement: Supplemental Information 1 — Supplementary figures/tables and code used for data analysis [file peerj-06-5486-s001.zip › Supplement/wolbachia_coverage_mapping/pdfs/Operophtera_brumata.pdf]

Total span assessed for coverage = 1.261 Mb  
Median coverage = 138.22

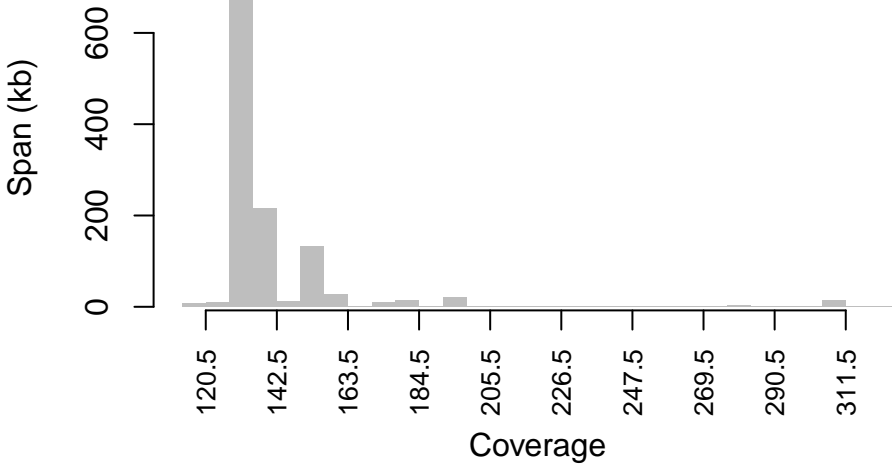

Supplement: Supplemental Information 1 — Supplementary figures/tables and code used for data analysis [file peerj-06-5486-s001.zip › Supplement/wolbachia_coverage_mapping/pdfs/Pararge_aegeria.pdf]

Total span assessed for coverage = 1.115 Mb  
Median coverage = 11.57

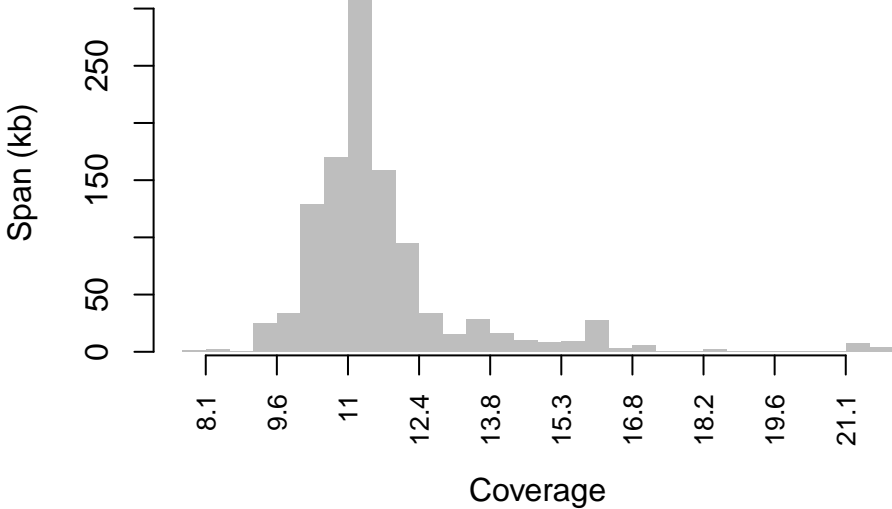

Supplement: Supplemental Information 1 — Supplementary figures/tables and code used for data analysis [file peerj-06-5486-s001.zip › Supplement/wolbachia_coverage_mapping/pdfs/Pediaspis_aceris_1.pdf]

Total span assessed for coverage = 1.098 Mb  
Median coverage = 11.60

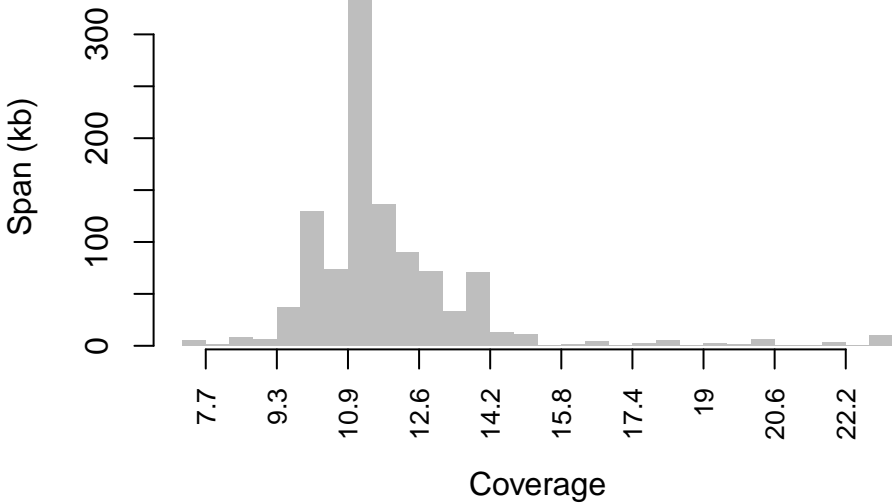

Supplement: Supplemental Information 1 — Supplementary figures/tables and code used for data analysis [file peerj-06-5486-s001.zip › Supplement/wolbachia_coverage_mapping/pdfs/Pediaspis_aceris_2.pdf]

Total span assessed for coverage = 1.368 Mb  
Median coverage = 60.81

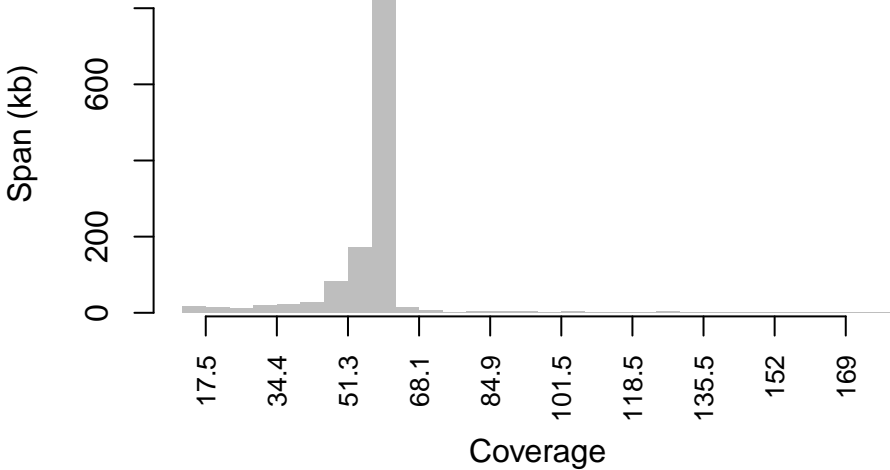

Supplement: Supplemental Information 1 — Supplementary figures/tables and code used for data analysis [file peerj-06-5486-s001.zip › Supplement/wolbachia_coverage_mapping/pdfs/Polygonia_c-album.pdf]

Total span assessed for coverage = 1.179 Mb  
Median coverage = 195.11

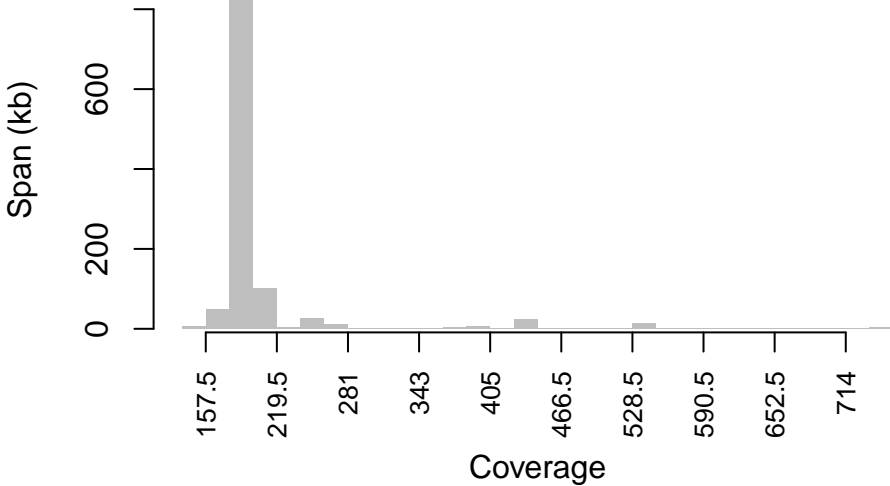

Supplement: Supplemental Information 1 — Supplementary figures/tables and code used for data analysis [file peerj-06-5486-s001.zip › Supplement/wolbachia_coverage_mapping/pdfs/Pseudomyrmex_sp_PSW_54.pdf]

Total span assessed for coverage = 1.192 Mb  
Median coverage = 267.94

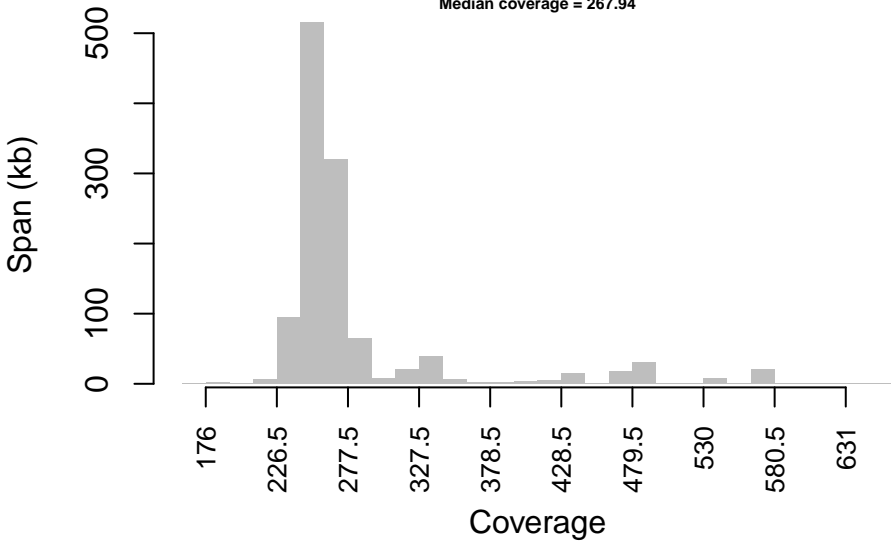

Supplement: Supplemental Information 1 — Supplementary figures/tables and code used for data analysis [file peerj-06-5486-s001.zip › Supplement/wolbachia_coverage_mapping/pdfs/Rhagoletis_pomonella.pdf]

Total span assessed for coverage = 1.754 Mb  
Median coverage = 1219.46

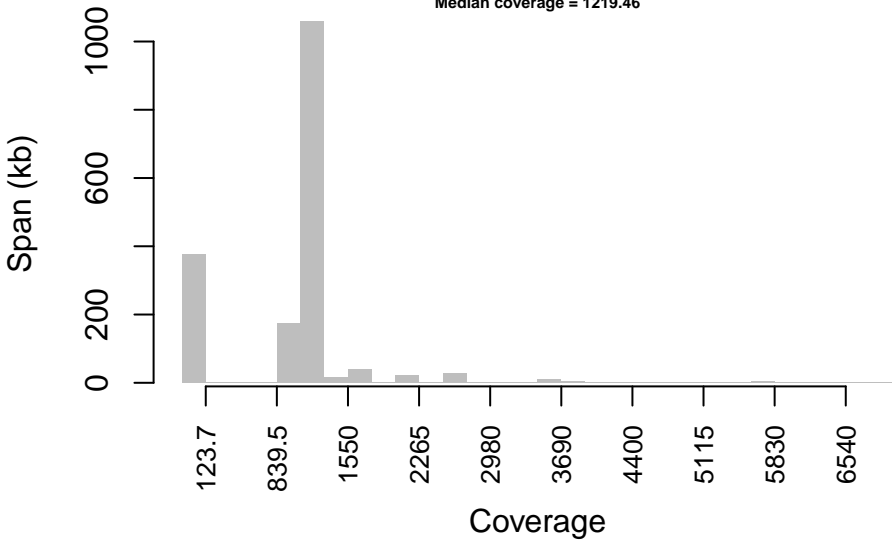

Supplement: Supplemental Information 1 — Supplementary figures/tables and code used for data analysis [file peerj-06-5486-s001.zip › Supplement/wolbachia_coverage_mapping/pdfs/Rhagoletis_zephyria.pdf]

Total span assessed for coverage = 1.075 Mb  
Median coverage = 50.37

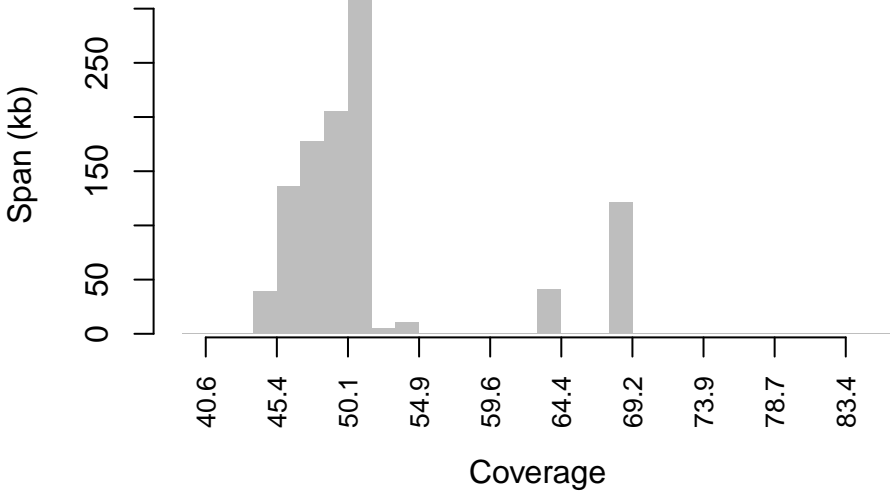

Supplement: Supplemental Information 1 — Supplementary figures/tables and code used for data analysis [file peerj-06-5486-s001.zip › Supplement/wolbachia_coverage_mapping/pdfs/Trichogramma_pretiosum.pdf]
